# IL-6 promotes MYC-induced B cell lymphomagenesis independent of STAT3

**DOI:** 10.1371/journal.pone.0247394

**Published:** 2021-03-02

**Authors:** Oleksi Petrenko, Jinyu Li, Velasco Cimica, Patricio Mena-Taboada, Ha Youn Shin, Stephen D’Amico, Nancy C. Reich

**Affiliations:** 1 Department of Microbiology and Immunology, Stony Brook University, Stony Brook, NY, United States of America; 2 Department of Pathology, Stony Brook University, Stony Brook, NY, United States of America; 3 American Type Culture Collection, City of Manassas, Virginia, United States of America; 4 University Frontera, Temuco, Chile; 5 Department of Biomedical Science & Engineering, Konkuk University, Seoul, Korea; Universitat des Saarlandes, GERMANY

## Abstract

The inflammatory cytokine IL-6 is known to play a causal role in the promotion of cancer, although the underlying mechanisms remain to be completely understood. Interplay between endogenous and environmental cues determines the fate of cancer development. The Eμ-*myc* transgenic mouse expresses elevated levels of c-Myc in the B cell lineage and develops B cell lymphomas with associated mutations in p53 or other genes linked to apoptosis. We generated Eμ-*myc* mice that either lacked the IL-6 gene, or lacked the STAT3 gene specifically in B cells to determine the role of the IL-6/JAK/STAT3 pathway in tumor development. Using the *Eμ-myc* lymphoma mouse model, we demonstrate that IL-6 is a critical tumor promoter during early stages of B cell lymphomagenesis. IL-6 is shown to inhibit the expression of tumor suppressors, notably BIM and PTEN, and this may contribute to advancing MYC-driven B cell tumorigenesis. Several miRNAs known to target BIM and PTEN are upregulated by IL-6 and likely lead to the stable suppression of pro-apoptotic pathways early during the tumorigenic process. STAT3, a classical downstream effector of IL-6, appears dispensable for *Eμ-myc* driven lymphomagenesis. We conclude that the growth-promoting and anti-apoptotic mechanisms activated by IL-6 are critically involved in *Eμ-myc* driven tumor initiation and progression, but the B cell intrinsic expression of STAT3 is not required.

## Introduction

The complex interplay between intrinsic genetic factors and externally driven signaling pathways is pivotal to cancer development. Chronic inflammation within a tumor microenvironment is frequently associated with high levels of the interleukin-6 [IL-6] cytokine, and IL-6 levels have been shown to steadily increase as cancers become life threatening [1–4]. IL-6 is produced by a broad range of cell types and has pleiotropic effects that include inflammation as well as regulation of metabolic, regenerative, and neural processes. A principal signaling mode of IL-6 involves activation of Janus tyrosine kinases [JAK] that are associated with the IL-6 receptor alpha chain and the common signal-transducing chain gp130 [5]. Tyrosine phosphorylation of the receptor creates docking sites for the STAT transcription factors and for SH2 domain adaptor proteins involved in activation of MAPK/ERK and PI3K/AKT signaling [6,7]. Abnormalities in these signaling nodes can drive cancer progression towards evasion of apoptosis, malignancy and treatment resistance.

Increased expression of IL-6 systemically and within the tumor microenvironment is attributed both to cancer cells and tumor-associated stromal cells [4,8]. IL-6 is known to exert many of its effects *via* activation of the ubiquitous transcription factors STAT3 and NF-kB, which both control the expression of anti-apoptotic, differentiation, and immune response genes. IL-6 signaling can thereby contribute to multiple aspects in the process of malignant progression, promoting cancer cell growth and enhancing the metastatic niche. Studies with murine cancer models and genetic deficiencies in IL-6 or STAT3 have substantiated their role in several different tumor types. IL-6 deletion in mice has been shown to impair B cell malignancies as well as colorectal, liver and pancreatic cancer [9–15]. In contrast, tissue-specific deletions of STAT3 have been found to either promote or inhibit tumorigenesis and metastasis in different experimental settings [12,14,16–24].

c-Myc [MYC] is one of the most frequently deregulated transcription factors in cancer [25]. Overexpression influences many signal pathways that coordinate cell growth and proliferation. To determine the influence of IL-6 on lymphomagenesis mediated by deregulated MYC expression, we used an *Eμ-myc* murine model of B cell lymphoma [26]. In this model, a transgene encoding MYC is controlled by the immunoglobulin heavy chain enhancer and thereby reprograms gene expression and accelerates B cell tumorigenesis. Chromosomal translocation of MYC with the immunoglobulin locus was first observed in Burkitt’s lymphoma resulting in constitutive MYC expression, and was the basis for generation of the *Eμ-myc* mice [26,27]. *Eμ-myc* lymphomas arise following acquisition of secondary mutations, including those that de-regulate tumor suppressor genes, such as p53 and ARF [28,29]. Suppression of the intrinsic apoptotic pathway is likewise implicated in *Eμ-myc* lymphomagenesis [30–33]. However, to date, few cooperative genetic mutations have been identified that underpin tumor onset [34–36]. Although the MYC transcriptome is extensive, the distinct pathways that cooperate with *Eμ-myc* to initiate and sustain global changes in cellular proliferation, metabolism, and senescence resistance remain to be clearly defined.

To identify signaling pathways that intersect and cooperate with MYC, we evaluated the contribution of the IL-6/STAT3 pathway to B cell development and cellular survival in the context of *Eμ-myc*-driven B cell lymphomagenesis. Ablation of the IL-6 gene in this murine model revealed several significant findings. *Eμ-myc* mice typically develop aggressive B-cell lymphomas at an early age, and loss of IL-6 delayed the development of these MYC-driven lymphomas. IL-6 upregulated the expression of miRNAs that inhibit pro-apoptotic pathways of BIM and PTEN, thereby promoting tumorigenesis. These changes appear to be stable and are notably reflected in the B cell lymphomas. Although the STAT3 transcription factor is a classical downstream mediator of IL-6 signaling, it was found to be dispensable for MYC-induced tumorigenesis in this genetic context.

## Materials and methods

### Mouse handling and euthanasia

This study was carried out in strict accordance with the recommendations in the Guide for the Care and Use of Laboratory Animals of the NIH. The protocol was approved by the Institutional Animal Care and Use Committee of Stony Brook University [IACUC #2011–0356]. The Division of Animal Laboratory Research at Stony Brook University operates in accordance with the American Association for Laboratory Animal Science [AALAS], the American College of Laboratory Animal Medicine [ACLAM], and Animal Welfare Assurance ID D16-00006 [A3011-01] of the National Institutes of Health [NIH]. Mice were housed in either standard filter topped shoebox microisolator cages or filtered individually ventilated cages. Rooms have 10–15 air changes per hour and are maintained at 70-72F. Pelleted irradiated Purina mouse chow [5053 or 5058] is provided ad lib and hyperfiltered water [2 micron] is provided via water bottles ad lib. All mice are provided with Envirodri nesting material. Long term singly housed animals may receive additional enrichment [paper tubes, nylabones, igloos] if consistent with study aims. Mice were euthanized with 3L/min carbon monoxide inhalation on the day that they met endpoint criteria, this method is consistent with the recommendations of the Panel on Euthanasia of the American Veterinary Medical Association [AVMA guidelines] and Stony Brook University IACUC. All personnel involved with animal handling completed online and in class animal training as required by Stony Brook University. Spontaneous tumors driven by the Eu-Myc gene normally develop by 6 months of age, but the lack of IL-6 required evaluation up to 12 months of age at which time all mice were euthanized. Mice were examined 2–3 times per week for the development of any swelling of lymph nodes both by visible examination and physical palpation. If detection of any tumor formation or any health issue, they were observed daily. Animals were euthanized with cumulative tumors less than 1cm^3^ estimated by caliper [volume = [width]^2^ x length/2], or if any signs of respiratory or behavioral distress. This included change in activity, posture, appearance, grooming, weight loss, movement or vocalization. Any tumor swelling or behaviors noted were the true endpoint of the experiment. Mice were euthanized with 3L/min carbon dioxide. There were no post-operative situations, and all efforts were made to minimize suffering. Three censored mice died prior to observation of endpoint criteria.

### Mice

*Eμ-myc* transgenic mice [B6.Cg-Tg[IghMyc]22Bri/J], *IL6* knockout mice [B6.129S2-Il6tm1Kopf/J], *TP53* knockout mice [B6.129S2-Trp53tm1Tyj/J], CD19-Cre transgenic mice [B6.129P2[C]-Cd19tm1[cre]Cgn/J] and LSL tdTomato transgenic mice [B6.Cg-Gt[ROSA]26Sortm14[CAG-tdTomato]Hze/J] were purchased from The Jackson Laboratory and bred to obtain the desired genotypes. STAT3 floxed mice were described previously [37]. All mice were maintained on the C57Bl/6 background. Mice were crossed to obtain the F2 and F3 generations of *IL6+/+* and *IL6-/- Eμ-myc mice; IL6+/+* and *IL6-/- Eμ-myc p53+/-* mice; *CD19-Cre Eμ-myc* mice, *CD19-Cre tdTomato Eμ-myc* mice; and *STAT3 flox/flox CD19-Cre tdTomato Eμ-myc* mice. Genotypes were verified by PCR amplification with primers specific for the corresponding WT and mutant alleles according to Jackson Laboratory and determined with DNA isolated from 1mm tail at the time of weaning. Athymic nude mice [strain code 491] were purchased from Charles River. For in vivo reconstitution assays, 3 million freshly isolated bone marrow or splenic B cells isolated by flow cytometry were injected into the tail veins of recipient mice in 0.1ml with a 27 g needle. Mice were monitored for tumor development and tumors were harvested from euthanized mice, weighed, and processed for histological examination and expression analysis as described [38].

### Flow cytometry

Fresh lymphoid tissue samples were examined or sorted by flow cytometry with antibodies to B220 [CD45R], CD19, CD43, IgM, IgD, CD4, CD8, CD144, and SCA1 [BD Pharmingen and eBioscience]. Isolation of B cells was performed with antibodies to both CD45R and CD19 and a BD FACSAria Cell Sorter. Sorting was supported by the Research Flow Cytometry Core Facility at Stony Brook University. For cell cycle analysis, lymphoid cells were fixed in 70% ethanol, stained with propidium iodide, and analyzed using FACSCalibur [BD] with CellQuest software.

### Expression and microarray analyses

RNA was isolated with a TRIzol reagent [Invitrogen] and RNA levels were measured by quantitative real-time RT-PCR relative to HPRT using whole-cell RNAs prepared from primary lymphoid cells or tumors. First-strand cDNA synthesis was performed using iScript cDNA synthesis kit and qPCR with the SsoAdvanced Universal and CFX96 Touch Real-time PCD detection system [BioRad]. The sequences of primers are available upon request. Preparation of cDNAs for microarray analysis and sample hybridization to Affymetrix miRNA Gene Chip 4.0 Array were performed using Affymetrix protocols of the Stony Brook University DNA Microarray Core Facility.

For protein analysis, whole cell extracts were prepared by lysing cells in buffer containing 10 mM TrisHCl, pH7.4, 150 mM NaCl, 1 mM EDTA, 10% glycerol, 1% Triton X100, 40 mM NaVO4, 0.1% SDS, and 1 x protease inhibitors [Roche]. Western blotting was performed using antibodies against MYC [N-262, Santa Cruz]; p19ARF [05–929, Upstate]; STAT3 [610190, BD], p53 [32532], STAT5 [9363], PIK3CA [C73F8], PTEN [D43], PDK1 [3062], AKT [9272], BCL-XL [2762], BIM [C34C5], P-ERK1/2 [4370] all from Cell Signaling; BCL2 [3498], and ERK1/2 [05–157] from EMD Millipore. Reverse phase protein array analysis [RPPA] was performed by the MD Anderson RPPA Core Facility.

### Statistical analyses

Kaplan-Meier and Log-Rank tests were used to compute and compare survival over time. Both male and female mice were used in the analyses and no significant sex difference was noted. Statistical analyses were performed using Student’s t test. P ≤ 0.05 was considered statistically significant. For the Affymetrix microarray analysis, the log2 values were supplied to the heat map function of the R statistical package [R-project.org]. The modified Gene Set Enrichment Algorithm was used to perform the pathway-based analyses. Module expression analysis was conducted as described [39]. Average gene expression values [log2] of all genes were set as baseline 0. The gene expression values [log2] of each module relative to the overall average were represented as mean ± SD. We used mirPath v.3 software for miRNA target prediction and pathway enrichment analysis [40]. RPPA data analysis was carried out using publicly available data sets [www.cbioportal.org] and the existing literature [39]. Pearson correlation coefficient was calculated with unsupervised cluster analysis and centroid linkage to visualize data [www.heatmapper.ca].

## Results

### IL-6 loss delays MYC-induced lymphomagenesis

**Fig 1:**
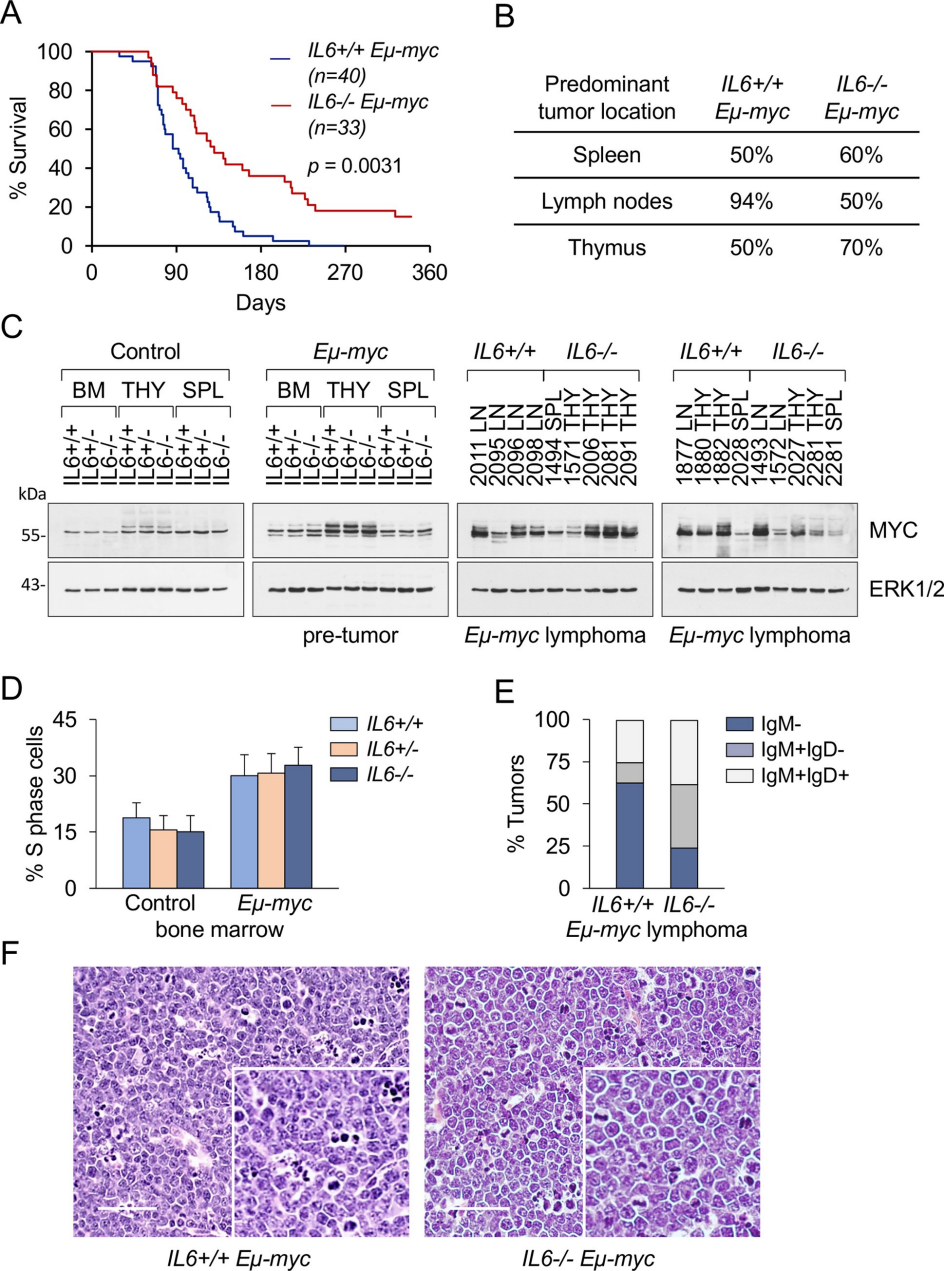
IL-6 loss delays Myc-induced B cell lymphomagenesis. **A.** Lymphoma survival of *IL6+/+* and *IL6-/-;Eμ-myc* mice. The survival of *Eμ-myc* mice with or without expression of the IL-6 gene is displayed graphically with Kaplan-Meier curves. The number of animals per genotype is indicated. The difference in the mean survival time of mice is statistically significant [p = 0.0031]. **B.** Predominant lymphoma localization. Autopsy findings of lymphomas in moribund *IL6+/+;Eμ-myc* [n = 16] or *IL6-/-;Eμ-myc* [n = 10] mice were quantified and results indicate differential patterns dependent on IL-6 genotype. **C.** Western blot analysis of MYC expression was measured in cells isolated from bone marrow [BM], thymi [THY], and spleens [SPL] of mice without the *Eμ-myc* transgene [control] or *Eμ-myc* pre-tumor [1 month age] mice of the indicated IL-6 genotypes, or in the lymphomas isolated from noted locations. ERK1/2 was used as an internal control. **D.** Comparative proliferation effects of IL-6. Flow cytometry was used to evaluate proliferation of bone marrow cells in 1 month old mice by cell cycle analysis with propidium iodide staining [FACSCalibur]. The percent of cells in S phase was measured in samples from 10 mice that lack the *Eμ-myc* transgene [control] or from 10 mice expressing the *Eμ-myc* transgene. The error bars represent the standard deviation. **E.** Immunophenotyping of lymphomas by flow cytometry from *IL6+/+;Eμ-myc* mice [n = 8] and *IL6-/-;Eμ-myc* mice [n = 8] with B220 and CD19 antibodies for B cell identity, and IgM and IgD antibodies for maturation. Tumors were classified as either pro-B [IgM-], immature B [IgM+IgD-] or mature [IgM+IgD+] B cell lymphomas. **F.** Histological examination [H&E staining] of representative lymphomas arising in *IL6+/+;Eμ-myc* and *IL6-/-;Eμ-myc* mice. Scale bars 100μM.

We evaluated the contribution of IL-6 in a spontaneous model of B cell lymphoma driven by the *Eμ-myc* transgene [26,38]. Cohorts of *IL6+/+;Eμ-myc*, *IL6+/-;Eμ-myc* and *IL6-/-;Eμ-myc* mice were generated and monitored for overall survival and tumor development. Whereas control *IL6+/+;Eμ-myc* mice developed lethal lymphomas with the expected latency [mean survival 103+/-46 days] and full penetrance [100% tumor incidence], the loss of IL-6 significantly delayed lymphomagenesis, extending tumor-free survival by 40% [164+/-99 days, p = 0.0031]. Moreover, up to 15% of mice remained tumor-free after 1 year of age [Fig 1A]. Histopathological analysis revealed that control *IL6+/+;Eμ-myc* mice developed advanced lymphomas with massive enlargement of lymph nodes [94% of the cases], spleen [50%] and thymus [50%], in agreement with previous reports [Figs 1B and S1C] [38]. In contrast, lymphadenopathy was not the prominent feature of *IL6-/-;Eμ-myc* lymphomas, as diseased mice displayed lymphoma mainly in the thymus and spleen [70% and 60% of the cases, respectively] [Figs 1B and S1B]. We found no differential effect of IL-6 on MYC protein expression in premalignant lymphoid tissues or in the *Eμ-myc* tumors [Fig 1C]. Likewise, there was no significant difference of IL-6 on proliferation of bone marrow cells in mice lacking the *Eμ-myc* transgene [control] or mice expressing the *Eμ-myc* transgene [Figs 1D and S1A] There was a distinction in the B cell differentiation stage of the *IL6+/+;Eμ-myc* and *IL6-/-;Eμ-myc* tumors. A majority of *IL6+/+;Eμ-myc* tumors were pro/pre-B cell lymphomas based on their IgM-negative immunophenotype [Fig 1E] and had a characteristic lymphoblastic morphology [Figs 1F and S1D]. In contrast, tumors arising in *IL6-/-;Eμ-myc* mice were markedly biased toward a more mature IgM+ B cell phenotype and were composed of large atypical lymphoid cells with large nuclei, resembling human diffuse large B cell lymphoma [DLBCL] [Figs 1F and S1D]. Thus, the loss of IL-6 extends the *Eμ-myc* lymphoma development to a later stage of B cell differentiation with a longer latency. These data are in line with previous studies of *Eμ-myc* mice showing that late onset lymphomas are distinct from early onset tumors reflecting the differences between Burkitt’s lymphoma and DLBCL [41].

### B cell development with loss of IL-6 in *Eμ-myc* mice

**Fig 2:**
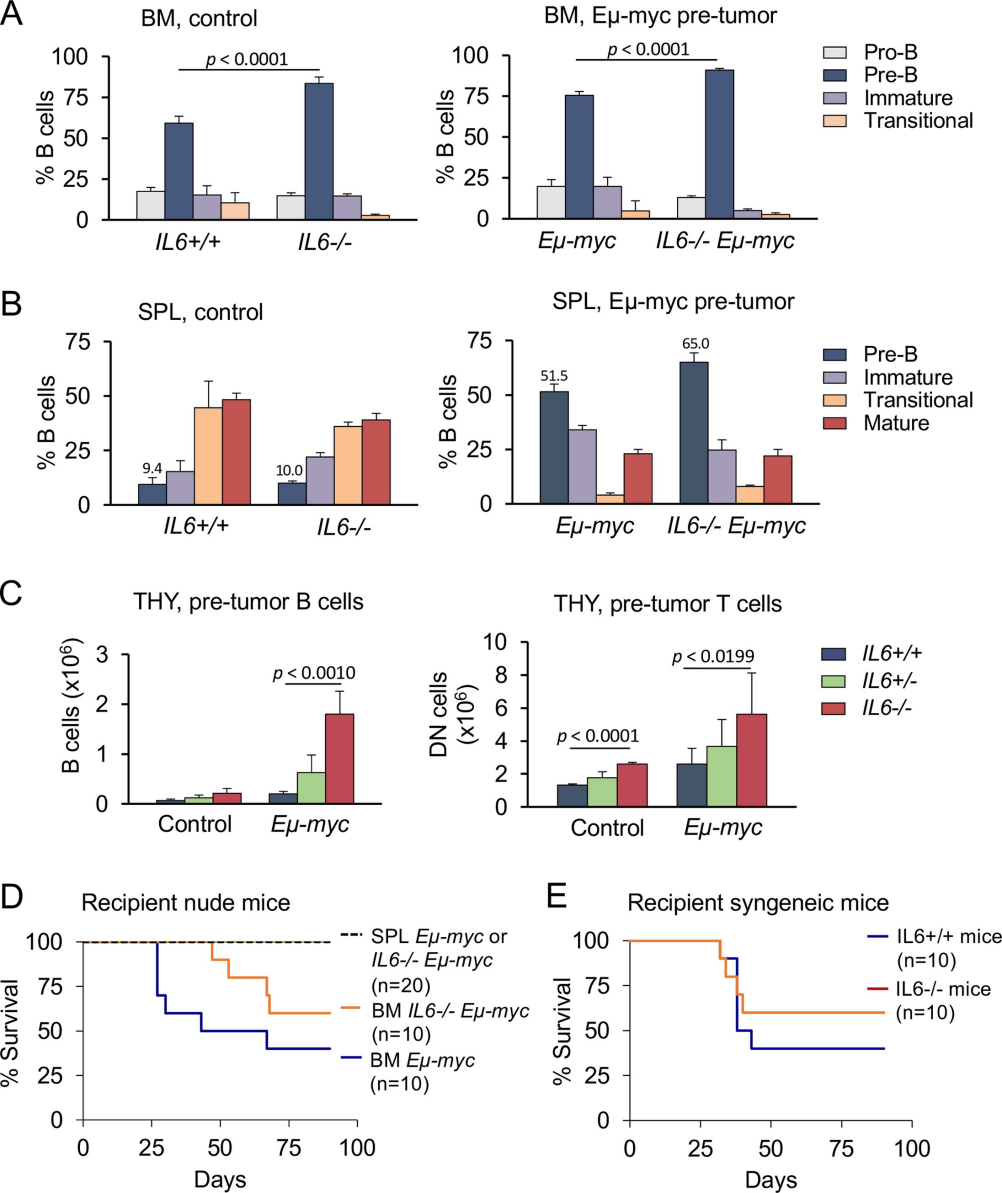
Inactivation of IL-6 does not perturb B cell development in mice. **A.** Flow cytometry analysis of B cells in the bone marrow [BM] and spleen [SPL] of 1 mo old *IL6+/+* [n = 6] and *IL6-/-* mice [n = 8]. Cell populations were defined as Pro-B [B220+CD43+IgM-], Pre-B [CD19+B220lowCD43-IgM-], immature B [CD19+B220lowIgM+], transitional B [CD19+B220highIgM+], and mature B [CD19+IgM+IgD+]. P value calculated from 6 mice. The error bars correspond to standard deviation. **B.** Flow cytometry analysis of B cells in the bone marrow [BM] and spleen [SPL] of 1 mo old *IL6+/+; Eμ-myc* [n = 6] and *IL6-/-;Eμ-myc* [n = 7] mice as described in [**2A**]. The error bars correspond to standard deviation and numbers represent the mean. **C.** Flow cytometry quantification of B cells with B220 and CD19 antibodies in the thymi of premalignant 1 mo old *Eμ-myc* mice of the indicated IL-6 genotypes [left]. Flow cytometry quantification of double negative [DN] T cells in thymi of premalignant mice of the indicated genotype [right]. Numbers of cells calculated per mouse/thymus. P values calculated from 6–8 mice. The error bars correspond to standard deviation. **D.** Bone marrow or splenic B cells from premalignant 1 mo old *IL6+/+;Eμ-myc* or *IL6-/-;Eμ-myc* mice were transplanted by tail vein injection into nude mice. Tumor development was monitored for a period of 90 days. Survival is displayed graphically with Kaplan-Meier curves. **E.** Bone marrow B cells from tumor-bearing *IL6-/-;Eμ-myc* mice were inoculated into syngeneic *IL6+/+* or *IL6-/-* mice by tail vein injection. Tumor development was monitored for a period of 90 days. Survival is displayed graphically with Kaplan-Meier curves.

To examine whether IL-6 loss affects B cell development, flow cytometry with specific differentiation markers was performed on whole bone marrow, spleen, and lymph nodes of 1-month old pre-tumor *Eμ-myc* mice and age-matched mice lacking the *Eμ-myc* transgene. Total cell numbers and B cell numbers in the bone marrow [BM], spleen [SPL], and lymph nodes were similar in *IL6-/-* and *IL6+/+* control [Figs 2A and S2A]. However, both *IL-6-/-* mice and *IL6-/-*:*Eμ-myc* young mice showed a modest increase in the percent of pre-B cells [CD19+B220lowCD43-IgM-] in bone marrow compared to mice expressing IL-6 [Fig 2A]. B cell development was not blocked with loss of IL-6, as B cells from *IL6-/-* mice exhibited normal immunophenotypes in the spleen and peripheral lymph nodes, consistent with previous reports [Figs 2B left, S2B and S2C] [42]. Although the bone marrow of young pre-malignant *IL6-/-*:*Eμ-myc* mice have an increase in immature pre-B cells, the B lymphomas that develop in adult *IL6-/-*:*Eμ-myc* mice have a mature phenotype in comparison with *IL6+/+*:*Eμ-myc* lymphomas [Fig 1].

The impact of *Eμ-myc* was evident in peripheral lymphatics regardless of the presence or absence of IL-6. Compared to *IL6-/-* and *IL6+/+* control mice, the spleen and lymph nodes of *Eμ-myc* mice had an increased percent of pre-B cells with a concordant reduction in transitional and mature B cells [Figs 2B, S2B and S2C]. Therefore, the delayed lymphomagenesis and enhanced survival of *IL6-/-;Eμ-myc* mice is not due to a reduction in early B cell numbers prior to disease onset. Notably, the high prevalence of tumors located in the thymi of *IL6-/-;Eμ-myc* mice [Fig 1B] was also reflected in a higher number of B cells in the thymi of pre-tumor *IL6-/-;Eμ-myc* mice compared to *IL6+/+;Eμ-myc* young mice [Figs 2C left, S2A and S2D]. The increase of *IL6-/-;Eμ-myc* in pre-tumor thymi may be due to altered homing signals associated with IL-6 deficiency. We also evaluated T cells in the thymi of pre-tumor mice since IL-6 is known to promote T cell differentiation. The *Eμ-myc* transgene increased the percent of immature double negative [DN] T cells in pre-tumor thymi, and this percent increased further in *IL6-/-;Eμ-myc* compared to IL6+/+;Eμ-myc mice [Figs 2C right and S2D] [43]. *Eμ-myc* expression reduced peripheral CD4 and CD8 T cells in pre-tumor spleens of both *IL6+/+;Eμ-myc* and *IL6-/-;Eμ-myc* mice [S2D Fig] [44].

To determine the influence of stromal and immune cells on development of B cell lymphomas, and to identify candidate B cells with tumor-initiating abilities, we performed adoptive transfer of B cells isolated from bone marrow or spleen into nude or syngeneic recipient mice. Donor B cells were isolated by fluorescence activated cell sorting [FACS] with CD19 and B220 antibodies. Nude mice received bone marrow or splenic B cells from 1-month old premalignant *IL6+/+;Eμ-myc* or *IL6-/-;Eμ-myc* mice by tail vein injection. Transplanted splenic [SPL] B cells from either *IL6+/+;Eμ-myc* or *IL6-/-;Eμ-myc* mice did not produce tumors in nude mice [Fig 2D]. However, adoptive transfer of bone marrow B cells maintained the lymphomagenic potential of the donor in that bone marrow B cells from IL-6+/+;*Eμ-myc* mice gave rise to tumors more rapidly in comparison to their IL-6-negative *Eμ-myc* counterparts [Fig 2D]. Therefore, there is an intrinsic difference in *IL6+/+;Eμ-myc* or *IL6-/-;Eμ-myc* early B cells that directs differential lymphomagenesis even in an immunodeficient recipient with greatly reduced T cells. In addition, the putative cell-of-origin of the B cell lymphomas in *Eμ-myc* mice appears to be an early B cell precursor prior to egress from the bone marrow.

To determine if the IL-6 stromal environment is a major contributor to the lymphomagenesis differences, we adoptively transferred B cells into *IL6+/+* or *IL6-/-* recipient mice. Adoptive transfer of bone marrow pre-tumor cells from 1 mo old mice into syngeneic wild-type mice was inefficient, presumably due to immune clearance and the generally low tumorigenic potential of early-stage *Eμ-myc* B cells [38,45]. For this reason we adoptively transferred bone marrow B cells derived from tumor-bearing *IL6-/-;Eμ-myc* mice into either *IL6+/+* or *IL6-/-* recipient mice. These transplanted tumor cells developed with similar efficiency in both IL-6-proficient and IL-6-deficient syngeneic recipient mice [Fig 2E]. These data imply that the onset of lymphomagenesis in *Eμ-myc* mice with different IL-6 genotypes is largely confined to cell autonomous bone marrow-derived B cell precursors.

### STAT3 is dispensable for MYC-mediated lymphomagenesis

**Fig 3:**
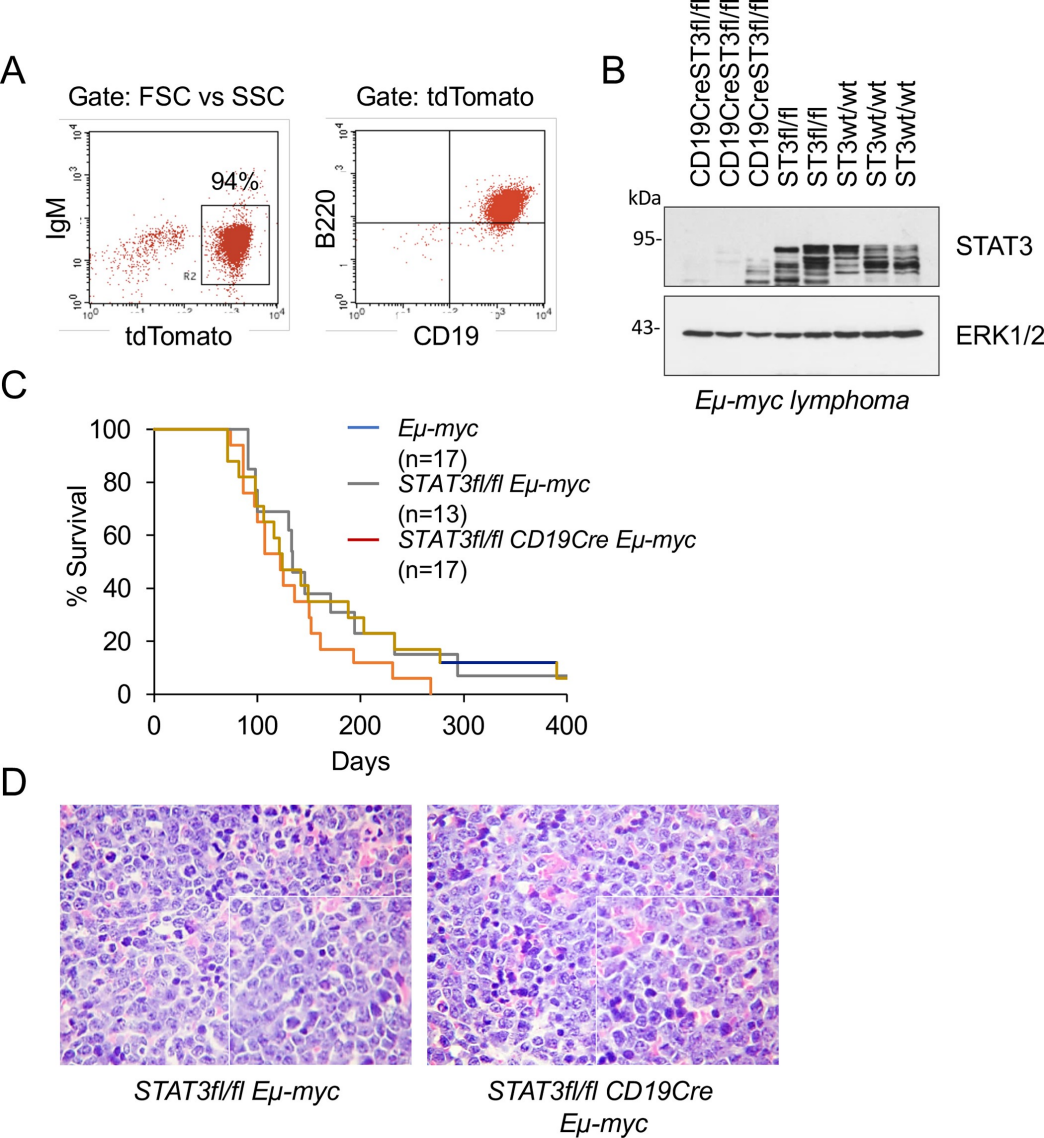
STAT3 is dispensable for MYC-mediated lymphomagenesis. **A.** Flow cytometry analysis of lymphoid tumors from *STAT3fl/fl;CD19Cre;LSL-tdTomato Eμ-myc* mice. Tumor cells were isolated and flow cytometry was used to access Cre recombinase efficiency by expression of tdTomato in cells stained with IgM antibodies [left] or B220 and CD19 antibodies [right]. **B.** Western blot analysis of STAT3 expression in tumors isolated from individual mice of genotype *STAT3fl/fl;CD19Cre;LSL-tdTomato Eμ-myc*, *STAT3fl/fl;LSL-tdTomato Eμ-myc* and *STAT3+/+ Eμ-myc*. ERK1/2 staining was used as a control. **C.** Comparative survival of *STAT3+/+;Eμ-myc* mice, *STAT3fl/flLSL-tdTomato;Eμ-myc* mice and *STAT3fl/flCD19Cre/+LSL-tdTomato;Eμ-myc* mice graphed with Kaplan-Meier curves. The number of animals used per genotype is indicated. **D.** Histological examination [H&E staining] of lymphomas from *STAT3fl/fl;Eμ-myc* mice and *STAT3fl/flCD19Cre/+;Eμ-myc* mice.

IL-6 activates multiple signaling pathways downstream of activated Janus kinases, most notably NF-kB and STAT3 [7,46–48]. NF-kB activity has been shown to be suppressed by MYC, and this suppression appears to be a prerequisite for *Eμ-myc* lymphomagenesis [49–51]. Therefore, we focused on STAT3 as a potential downstream mediator of IL-6 action in *Eμ-myc* lymphomas. The STAT3 genetic knockout is embryonic lethal, and for this reason we generated mice with a specific deletion of STAT3 in B cells with the Cre-Lox recombination system regulated by B cell-specific CD19 promoter [*STAT3fl/fl;CD19Cre*] [37,52,53]. Prior studies showed that STAT3 is efficiently deleted in the B cell lineage of *STAT3fl/fl;CD19Cre* mice, and that STAT3 is dispensable for the early stages of B cell development in mice [54]. STAT3 deletion in B cells does not impair germinal center responses for immunoglobulin class switching in the spleen, or antibody titers to a viral pathogen [55]. To monitor the efficiency of the Cre recombinase in the B cells, we additionally crossed *STAT3fl/fl;CD19Cre Eμ-myc* mice with *Rosa-CAG-LSL-tdTomato* mice [56]. In all cases examined, the efficiency of recombination and *tdTomato* expression in B cell lymphomas was >90%, as measured by flow cytometry and Western blotting [Fig 3A and 3B]. The impact of STAT3 on lymphomagenesis in *Eμ-myc* mice was evaluated by comparison of tumor development in *STAT3wt/wt;Eμ-myc*, *STAT3fl/fl;tdT;Eμ-myc*, *and STAT3fl/fl;tdT;CD19Cre;Eμ-myc* mice [Fig 3C]. We observed that STAT3 loss in B cells did not affect tumor incidence, severity, differentiation, or overall survival of *Eμ-myc* mice [Fig 3C and 3D]. STAT3 protein expression did not notably differ between *IL6+/+;Eμ-myc* and *IL6-/-*;*Eμ-myc* premalignant lymphoid tissues or tumors [S3A Fig]. We therefore conclude that the presence or absence of IL-6 impacts MYC function through mechanisms that are distinct from STAT3 activation.

### Targeted inactivation of p53 dominantly accelerates *Eμ-myc* lymphomas

**Fig 4:**
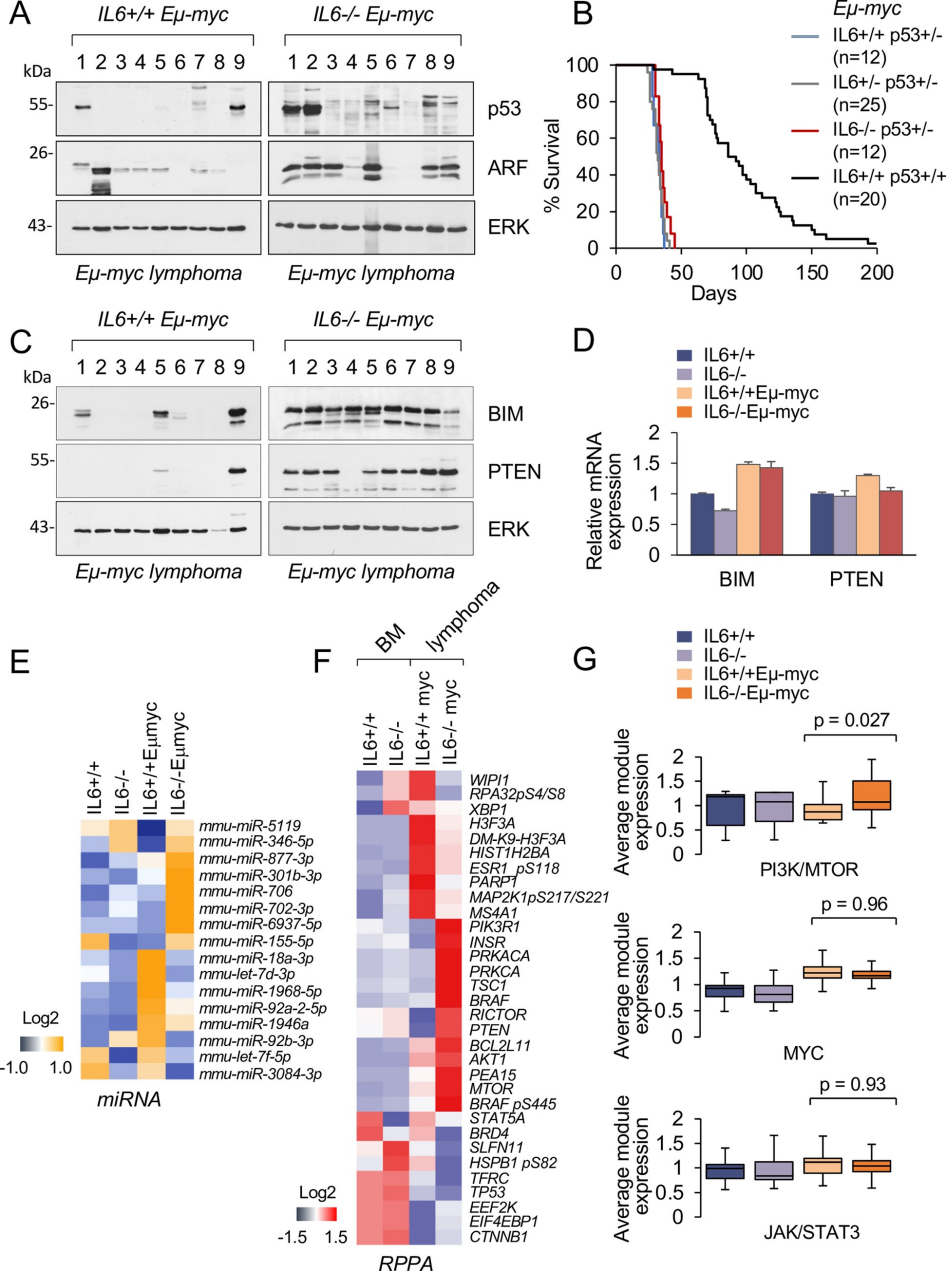
IL6 suppresses pro-apoptotic pathways in *Eμ-myc* mice. **A.** p53 and ARF protein expression was measured in B cell lymphomas isolated from individual mice. Western blot analysis was performed with lymphomas derived from IL6+/+;*Eμ-myc* and IL6-/-;*Eμ-myc* mice. ERK1/2 staining was used as a control. **B.** Survival of mice with the *Eμ-myc* transgene and deficiency for IL-6 and/or p53 was measured and graphed by Kaplan-Meier curves. The distinct genotypes and the number of mice are shown. **C.** Lymphomas were derived from individual *IL6+/+;Eμ-myc* and *IL6-/-;Eμ-myc* mice, and BIM and PTEN protein expression was measured by Western blot. ERK1/2 staining was used as a control. **D.** Real-time quantitative PCR analysis of BIM and PTEN mRNA expression in bone marrow cells of *IL6+/+*, *IL6-/-*, *IL6+/+;Eμ-myc* and *IL6-/-;Eμ-myc* mice. 2–3 independent samples were processed for each genotype with HPRT mRNA used as control. **E.** Heat map representing differentially expressed miRNAs in bone marrow-derived pre-tumor B cells [1 mo old] from *IL6+/+*, *IL6-/-*, *IL6+/+;Eμ-myc*, and *IL6-/-;Eμ-myc* mice. B cells were isolated by FACS from bone marrows, RNA was prepared, and cDNA synthesized was hybridized to Affymetrix miRNA 4.0 Gene Chip Arrays and analyzed by the Stony Brook University Core Facility. Cells were pooled from 3–5 mice of each genotype. **F.** Differentially expressed proteins are represented as heat maps of reverse phase protein array [RPPA] data from 8–10 pooled samples of bone marrow B cells from *IL6+/+* and *IL6-/-* mice, or B lymphomas from *IL6+/+;Eμ-myc* and *IL6-/-;Eμ-myc* mice. **G.** Perturbation of the PI3K/MTOR, MYC and JAK/STAT3 signaling modules caused by loss of IL-6 function. Average protein expression levels are shown derived from RPPA.

Evasion of apoptosis is a critical characteristic of MYC-induced tumorigenesis, and one well-characterized factor known to promote apoptosis is the p53 tumor suppressor. Lymphomas arising in *Eμ-myc* mice harbor frequent loss of function mutations in the TP53 and CDKN2A tumor suppressors, leading to markedly increased p53 and ARF protein levels [28,57]. Western blot analysis revealed that gross p53 and ARF overexpression was more frequent in *IL6-/-;Eμ-myc* lymphomas [~80% of cases] as compared with *IL6+/+;Eμ-myc* lymphomas [~40% of cases] suggesting that tumorigenesis in IL-6-deficient mice may specifically select for a loss of p53 function. Individual lymphoma examples are shown in Fig 4A. The frequency of p53 inactivation/ARF overexpression in *IL6+/+;Eμ-myc* tumors is consistent with previous studies [28,57].

To determine whether p53 inactivation could accelerate the development of *IL6-/-;Eμ-myc* lymphomas and produce tumors that are phenotypically similar to those with intact IL-6, we examined lymphoma onset and latency in genetically matched *IL6+/+;Eμ-myc;p53+/-* and *IL6-/-;Eμ-myc;p53+/-* mice. It has been shown that lymphomas arising in *p53+/-* mice invariably lose the wild-type p53 allele and become p53-null [28,57]. In this context, both IL-6-proficient and IL-6-deficient *Eμ-myc;p53+/−* mice developed lymphomas more rapidly, by 5 weeks of age, than mice with wild-type p53 alleles [Fig 4B]. Therefore, targeted disruption of p53 dominantly accelerates *Eμ-myc* tumorigenesis with or without IL-6 expression.

### IL-6 suppresses pro-apoptotic pathways in *Eμ-myc* mice

Because apoptosis and tumor suppression sensitized by MYC can be p53-independent [58], we examined expression of other genes controlling B cell survival and lymphomagenesis. Two of the genes evaluated showed elevated protein expression in tumors from IL-6-deficient *Eμ-myc* mice: BIM [BCL2L11], a BCL-XL and BCL2 interacting mediator of cell death, and PTEN, a phospholipid phosphatase and tumor suppressor gene mutated in many human cancers [Fig 4C]. Prior to the onset of frank lymphoma, low variable levels of BIM and PTEN were expressed in *Eμ-myc* cells of both IL-6 genotypes [S3B Fig]. As lymphomas arose, enhanced expression of both BIM and PTEN accompanied ~90% of tumors arising in *IL6-/-;Eμ-myc* mice [Fig 4C]. In contrast, BIM expression increased in only 40% [8/20] of *IL6+/+;Eμ-myc* tumors and PTEN expression increased in only 20% [4/20] of *IL6+/+;Eμ-myc* tumors. Lymphoma examples are shown in Fig 4C]. The lower levels of BIM and PTEN potentially contribute to the survival of *IL6+/+;Eμ-myc* tumor cells. Tumors deficient in p53 retain BIM and PTEN expression indicating that inactivation of either the p53 or PTEN/BIM pathway promotes lymphoma development [S4A Fig]. Furthermore, *IL6-/-;Eμ-myc* lymphomas transplanted into syngeneic *IL-6+/+* or *IL6-/-* mice retained PTEN and BIM expression [S4B Fig]. These findings support an essential cell autonomous role of IL-6 in mediating the anti-apoptotic and tumor-promoting activity of MYC.

To determine whether the increase in BIM and PTEN protein expression in *IL-6-/-;Eμ-myc* lymphoma cells reflected an increase in their mRNA expression, we compared mRNA levels. RNA was isolated from bone marrow or lymphomas and samples were pooled from each genotype and evaluated by quantitative real time PCR analyses. Unexpectedly the levels of PTEN and BIM mRNAs did not show a significant increase in *IL6-/-;Eμ-myc* compared to *IL6+/+;Eμ-myc* samples [Fig 4D]. A principle effect of MYC activation in *Eμ-myc* mice promotes widespread changes of microRNA [miRNA] expression. For instance, dysregulation of miR-17-92 is thought to play a causative role in *Eμ-myc* lymphomagenesis by coordinating multiple oncogenic pathways [59,60]. To determine if BIM and PTEN could be targets of IL-6 regulation by miRNA expression, we examined the expression levels of >800 miRNAs with the Affymetrix Gene Chip miRNA 4.0 array. RNA samples were prepared from BM-derived B cells from 1-month old premalignant *IL6+/+;Eμ-myc* mice and *IL6-/-;Eμ-myc* mice, and *IL6+/+* and *IL6-/-* controls. We reasoned that the pre-tumor setting rather than the MYC-driven lymphoma setting may more faithfully reflect the role of IL-6 in the earliest steps of *Eμ-myc* tumorigenesis. The analysis revealed a high degree of similarity between *IL6+/+* and *IL6-/-* B cells, but significant differences between these control cells and the *IL6+/+;Eμ-myc* and *IL6-/-;Eμ-myc* mice [Fig 4E]. More than 40 miRNAs were deregulated [up or down] with a fold change of four or more in the respective *Eμ-myc* B cells at premalignant stages. Significantly, several of the miRNAs known to target BIM and PTEN [e.g. *mmu-miR-18a-3*, *mmu-miR-92a-2-5p* and *mmu-miR-92b-3p*] are upregulated in *IL6+/+;Eμ-myc* B cells as compared to their *IL6-/-;Eμ-myc* counterparts, and presumed to contribute to the BIM and PTEN suppression in *IL6+/+;Eμ-myc* lymphomas [Fig 4E] [61–64]. To examine the expression of tumor suppressor genes with that of IL-6 expression in human B lymphomas, we analyzed the mRNA expression data available in a TCGA lymphoid DLBCL dataset [PanCancer Atlas]. Expression of the IL-6 gene and the PTEN, BCL2L11, TP53, and CDKNA2 genes was evaluated [S5 Fig]. Although the samples and data are limited, the trend is an inverse correlation of IL-6 RNA expression with these pro-apoptotic tumor suppressor genes, in support of our interpretations with IL-6 in *Eμ-myc* mice.

To further characterize protein expression dependent on IL-6 and MYC, we evaluated global protein expression profiles in bone marrow of *IL6+/+* and *IL6-/-* mice as well as in lymphomas from *IL6+/+;Eμ-myc* and *IL6-/-;Eμ-myc* mice [Fig 4F]. To minimize individual mouse variation, protein lysates representing normal or tumor samples of each genotype were pooled prior to evaluation by reverse phase protein array [RPPA] analysis. *Eμ-myc* lymphomas are inherently heterogeneous, usually reflected by chromosomal aberrations, gene expression changes and differences in the latency of disease [41,65]. Even with expected heterogeneity, the RPPA analysis showed clear distinctions in the samples reflecting the dominant role of MYC [S6A Fig]. We identified more than 30 differentially expressed proteins in *IL6-/-;Eμ-myc* tumors compared with the *IL6+/+;Eμ-myc* counterparts, including the increased expression of BIM [BCL2L11] and PTEN [Fig 4F]. The expression of select proteins was confirmed by Western blotting [S6B–S6D Fig]. Analysis of the differentially abundant proteins in *IL6+/+;Eμ-myc* and *IL6-/-;Eμ-myc* samples revealed that the majority fall into the functional categories of the PI3K/AKT/MTOR signaling network [e.g., AKT1 and MTOR; PTEN and TSC1] [Fig 4G] [39]. There were no significant changes in MYC or JAK/STAT3 expression modules. This result is significant in view of the fact that deregulation of the PI3K/AKT/MTOR cooperates with MYC in bypassing senescence during the course of *Eμ-myc* lymphomagenesis [66]. The results suggest that development of B lymphomas in the absence of IL-6 is more dependent on the MTOR pathway for tumorigenesis or evasion of pro-apoptotic signals than in the presence of IL-6.

## Discussion

Recurrent MYC translocations, either alone or in combination with BCL2 rearrangements, are the hallmark of Burkitt’s lymphoma and other mature B-cell neoplasms that are usually associated with an aggressive clinical behavior [67]. Like most human cancer models driven by MYC, the development of lymphomas in *Eμ-myc* mice involves the subversion of normal signaling pathways and the acquisition of secondary driver mutations, resulting in clonal expansion of tumor cells. One reflection of this dependency in *Eμ-myc* mice may be seen in the variable time of tumor onset, low rate at which the benign B cells convert to malignancy [~10^−10^ per cell per generation], and the presence of recurrent mutations in cancer-related genes, such as RAS, TP53 and CDKN2A [28,34,35,57]. Disruption of pro-apoptotic BCL2 family genes [e.g., BAD, BIM, BMF, PUMA] also accelerates lymphoma development in the *Eμ-myc* model [30–33]. However, whether mutations affecting these genes play causal roles in spontaneous lymphoma and leukemia of early B cells remains to be determined.

To define the functional interface between MYC and IL-6 in lymphomagenesis, a comprehensive biochemical and functional examination of *Eμ-myc*-driven lymphomas arising in wild-type and IL-6 knockout [*IL6−/−*] mice was performed. Evidence is provided that the inflammatory cytokine IL-6 is a critical tumor promoter during early stages of *Eμ-myc*-driven B cell lymphomagenesis. *Eμ-myc* mice typically develop immature pro/pre-B cell lymphomas at an early age. In contrast, the loss of IL-6 generated *Eμ-myc* lymphomas at a later stage of B cell differentiation [DLBCL-like], and with a longer latency. Although the *IL6-/-;Eμ-myc* lymphomas were comprised of more mature B cells, pre-tumor bone marrows showed an increase in pre-B immature cells compared with *IL6+/+;Eμ-myc* mice [Fig 2]. Future studies are needed to determine whether this modest alteration in development influences tumorigenic properties, and if the shorter half-life of immature B cells contributes to a delay in accumulation of secondary oncogenic mutations leading to mature tumors and longer latency.

We describe a fundamental and previously unexplored IL-6-dependent mechanistic pathway underlying induction of spontaneous B-cell lymphomas. Our data indicate that IL-6 signaling enhances the resistance of B cells to apoptosis and thereby diminishes the *requirement for pro-apoptotic gene* mutations during tumorigenesis. While *Eμ-myc* mice typically develop B-cell lymphomas at an early age, the loss of IL-6 affects gene expression changes which promote apoptosis or growth arrest in premalignant cells, thereby reducing tumorigenesis. Accordingly, the loss of IL-6 selects for *Eμ-myc* lymphomas at a later stage of differentiation and with a longer latency. Tumors arising in *IL6-/-;Eμ-myc* mice are markedly biased toward a more mature IgM+ B cell phenotype and are composed of large atypical lymphoid cells, resembling diffuse large B cell lymphoma [DLBCL], the most common non-Hodgkin lymphoma. We find that the changes induced by IL-6 loss are stable and appear to occur early during tumorigenic conversion of precancerous cells. These data are in line with previous studies of *Eμ-myc* mice that found lymphoma latency to be influenced by IL-6 signaling [68] and that late onset lymphomas are distinct from early onset tumors reflecting distinctions between Burkitt’s lymphoma and DLBCL [41]. Studies with human DLBCL cell lines have shown autocrine IL-6 and JAK1 signaling promote DLBCL viability [15,69]. The DLBCL that develops independent of IL-6 signaling may represent patient subtypes with better prognosis [70,71].

The molecular mechanisms of cooperation between IL-6 and MYC in lymphomagenesis remained to be completely understood. STAT3 is a classical downstream effector of IL-6, but we find that it is dispensable for *Eμ-myc* driven lymphomagenesis. Cre-mediated deletion of STAT3 in *Eμ-myc* B cells did not alter the development of lymphomas. We conclude that the growth promoting and anti-apoptotic mechanisms activated by IL-6, but not IL-6-mediated STAT3 activation, are critically involved in *Eμ-myc* driven tumor initiation and progression. DLBCL studies have shown a JAK1 requirement for viability that cannot be compensated by STAT3 activity [69]. The slower development of *Eμ-myc* lymphomas deficient in IL-6 may rely on activation of non-STAT pathways such as MAPK/ERK and PI3K/AKT, or STAT pathways activated by other cytokines.

A significant finding of our study is that IL-6 cooperates with MYC by promoting the repression of tumor suppressors BIM and PTEN. The expression changes induced by IL-6 are stable and occur early during tumorigenic conversion of precancerous cells. Results indicate that the IL-6-dependent repression of BIM and PTEN in *Eμ-myc* mice is coordinate with induction of the regulatory miR-17-92 locus [62,72]. The miR-17-92 locus encodes a number of miRNAs including miR-18a, miR-92a, and miR-92b that are known to inhibit BIM and PTEN in experimental models of cancer [61,63,64]. These miRNAs were found to be induced in pretumor BM cells of *IL6+/+;Eμ-myc* mice but not *IL6-/-;Eμ-myc* mice [Fig 4E]. Hyperactive MYC increases expression of the miR-17-92 cluster [73], and this study indicates IL-6 is required to act in concert with MYC to increase transcription of mir-17-92. The encoded miRNAs in turn could reduce BIM and PTEN expression, contributing to B cell survival and proliferation.

Our data also imply that there is redundancy and functional compensation in B-cell lymphomas between p53 and the BIM and PTEN tumor suppressors. The majority of tumors arising in *IL6-/-;Eμ-myc* mice, but not *IL6+/+;Eμ-myc* mice, tend to retain BIM and PTEN expression, while the majority of tumors arising in *IL6-/-;Eμ-myc* mice, but not *IL6+/+;Eμ-myc* mice, tend to inactivate p53 [Fig 4]. Indeed, mutations of PTEN and p53 in cancer are frequent yet often mutually exclusive, in part because PTEN and p53 regulate each other’s levels and activity [74]. In addition, the p53 pathway was found to be unaffected in most BIM-deficient *Eμ-myc* tumors, providing evidence that BIM reduction is an alternative to loss of p53 [30]. IL-6 signaling appears to tip the balance between these tumor suppressor pathways in favor of p53. The TCGA lymphoid DLBCL dataset [PanCancer Atlas] of RNA expression indicates an inverse relationship between IL-6 expression and that of PTEN, BCL2L11, TP53, and CDKNA2 [S5 Fig]. The loss of IL-6 in MYC lymphomas also leads to a compensatory increase in proteins expressed in the mTOR pathway [75,76]. A reciprocal relationship between IL-6 and PI3K/ MTOR pathway has been proposed previously [77]. The data indicate that without IL-6, the Eμ-myc lymphomas are more dependent on the mTOR pathway. The mTOR signaling axis may be activated due to the cellular stress of increased BIM and PTEN countered with the effects of MYC.

## Conclusions

Knowledge of the functional interface between the pro-oncogenic factor MYC and the IL-6 cytokine is critical to understand B lymphoma development and responses to therapeutic strategies. We demonstrate in a spontaneous murine model of B lymphoma [*Eμ-myc*] that IL-6 promotes lymphomagenesis without the need of downstream intrinsic signaling by the STAT3 transcription factor. In addition, our data suggest the differential expression of non-coding RNAs that regulate pro-apoptotic pathways may be key to understanding IL-6-mediated survival of MYC-driven B cell lymphoma.

## Supporting information

S1 Fig Effects of IL-6 loss on survival and tumor phenotype. **A.** IL-6 does not have a differential effect on S phase distribution of bone marrow or splenic cells. Cells were isolated from 1 month old mice without the *Eμ-myc* transgene or with the transgene and the indicated IL6 genotypes [n = 6 for each genotype]. The percent of cells in S phase was measured by cell cycle analysis with propidium iodide staining and flow cytometry [FACSCalibur]. The error bars represent the standard deviation. **B.** Average tumor weights. Tumors formed in lymph nodes, thymi and spleens were isolated and weighed from moribund mice of the indicated genotypes [n>10 for each genotype]. The error bars represent the standard deviation. **C.** Representative moribund *IL6+/+* and *IL6-/-;Eμ-myc* mice are shown. Dashed line circles reflect presence [in *IL6+/+;Eμ-myc]* or absence [in *IL6-/-;Eμ-myc*] of enlarged lymph nodes. Solid line circle demarks lymphoma in thymus of *IL6-/-;Eμ-myc* mouse. **D.** Histological examination [H&E staining] of lymphomas arising in *Eμ-myc* mice of the indicated IL-6 genotypes. Scale bars 100μM.
(PDF)

S2 Fig B cell development in the absence of IL-6. **A.** Total cellularity of primary and secondary lymphoid organs as noted of 1-month old control and *Eμ-myc* mice of the indicated IL-6 genotypes [n>10 for each genotype]. The error bars correspond to standard deviation. **B.** Flow cytometry analysis of B cells in the spleen and lymph nodes of 1-month old non-transgenic *IL6+/+* [WT] and *IL6-/-* mice. Cell populations were defined as Pre-B [CD19+B220lowCD43-IgM-], immature B [CD19+B220lowIgM+], transitional B [CD19+B220highIgM+], mature B [CD19+IgM+IgD+]. follicular B [FOB] [B220+IgMlow/-IgD+] and marginal zone B [MZB] [B220+IgM+IgDlow/-] [78]. The results are representative of 6 to 8 mice of each genotype. The error bars correspond to standard deviation. **C.** Flow cytometry analysis of B cells in the spleen and lymph nodes of 1-month old *IL6+/+; Eμ-myc and IL6-/-;Eμ-myc* mice as in **S2B**. **D.** Representative flow cytometry scatter plots are shown of thymi or spleens from mice with specific genotypes. *IL6-/- Eμ-myc* mice showed elevated B cell numbers in pre-tumor thymi [top left]. *IL6-/- and IL6-/- Eμ-myc* mice showed increased immature double negative T cells in pre-tumor thymi [bottom left]. *Eμ-myc* mice had reduced CD4+ and CD8+ T cells in pre-tumor spleens, particularly evident in *IL6-/- Eμ-myc* mice.
(PDF)

S3 Fig Protein expression in lymphoid tissue. **A.** Western blot analysis of STAT3, STAT5, and STAT1 expression in bone marrow [BM], thymi [THY], and spleens [SPL] of individual control mice lacking the *Eμ-myc* transgene or pre-tumor [1 mo age], of *Eμ-myc* mice with the indicated IL-6 genotypes, and in B cell lymphomas derived from these mice. **B.** Western blot analysis of p53 and ARF expression in BM, THY and SPL of control mice lacking the *Eμ-myc* transgene, and of pre-tumor [1 mo age] *Eμ-myc* mice, and B cell lymphomas derived from these mice.
(PDF)

S4 Fig Alterations in PTEN and BIM protein with IL-6 loss. **A.** Western blot of PTEN and BIM expression in BM from individual mice *IL6+/+;p53+/- Eμ-myc* and *IL6-/-;p53+/- Eμ-myc*. **B.** Western blot analysis of PTEN and BIM expression in *IL6-/-;Eμ-myc* lymphomas developed from cell transplants into WT or IL6-/- syngeneic recipients. [C] corresponds to sample of bone marrow from a control mouse that received no transplant.
(PDF)

S5 Fig Relative expression of IL-6 gene and four tumor suppressor genes in Diffuse Large B-Cell Lymphomas [DLBCL] in a human TCGA dataset, PanCancer Atlas. The heatmap displays mRNA expression of the IL-6 gene and the PTEN, BCL2L11, TP53, and CDKNA2 genes in 48 DLBCL samples. Z-scores relative to diploid samples [RNA Seq V2 RSEM] are presented [cBioportal.org] [heatmapper.ca]. A scatter plot comparing IL-6 expression with the sum of Z-scores of the four tumor suppressor genes [tumor suppressor signature, TSS] is shown for each sample.
(PDF)

S6 Fig Affymetrix miRNA analyses and supportive protein expression. **A.** Western blot analyses of primary and secondary lymphoid organs of 1 month old *Eμ-myc* mice of the indicated IL-6 genotypes. **B.** Western blot of BM protein samples from a subset of the individual *IL6+/+;Eμ-myc* and *IL6-/-;Eμ-myc* mice that were pooled for the RPPA analyses. **C.** Western blot for PTEN and BIM in BM samples from pre-tumor mice and tumor-bearing *IL6+/+;Eμ-myc* and *IL6-/-;Eμ-myc* mice.
(PDF)

S1 Raw images (PDF)
