# Supplementary figures and images for: IL-6 promotes MYC-induced B cell lymphomagenesis independent of STAT3
Source: PLoS One. 2021 Mar 2;16(3):e0247394. doi: 10.1371/journal.pone.0247394 (PMC7924759; doi:10.1371/journal.pone.0247394)

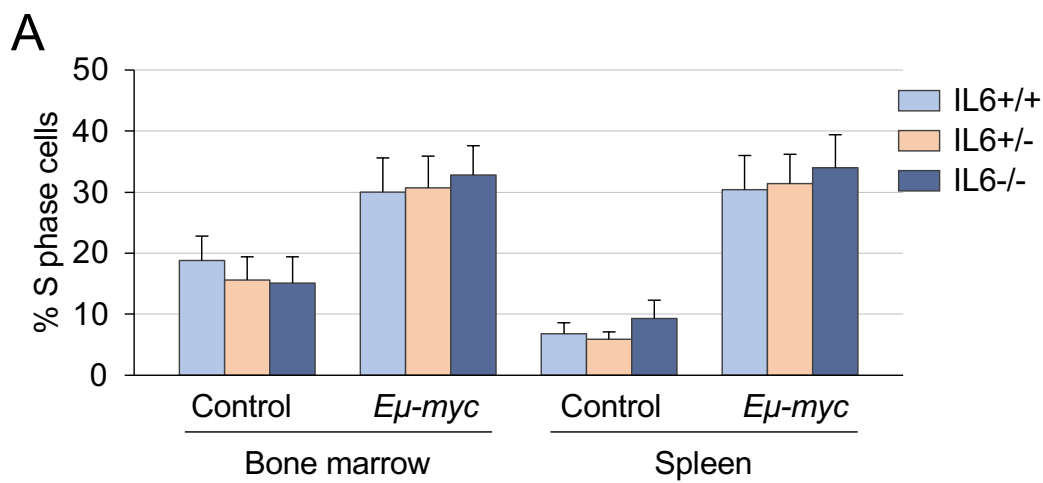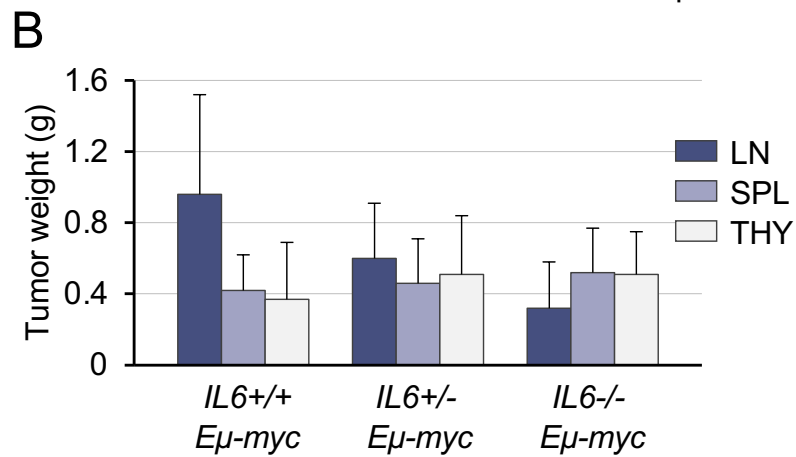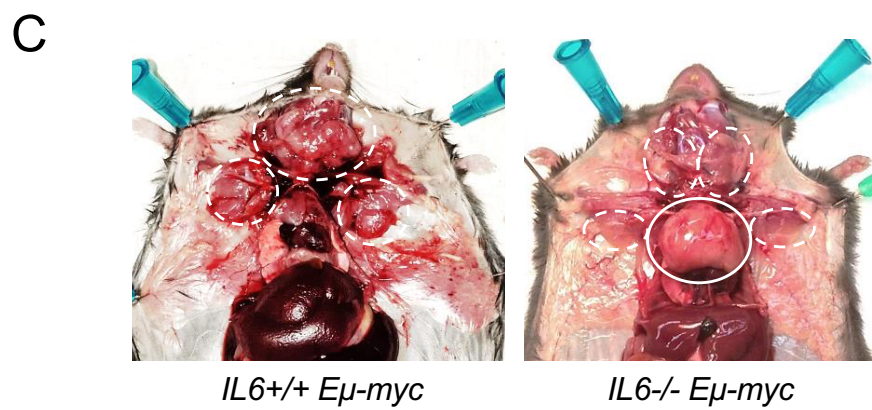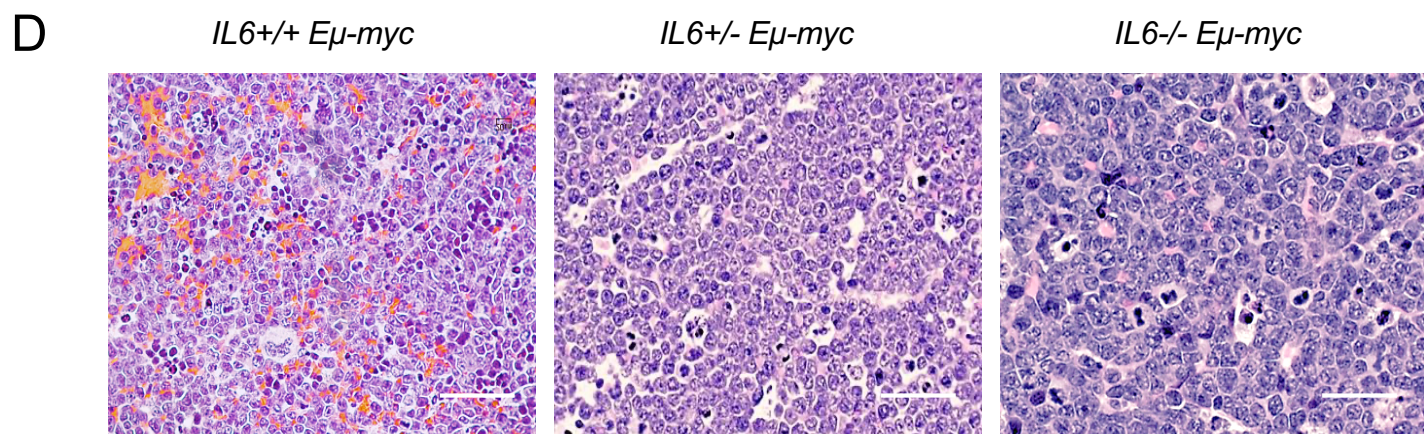

Supplement: S1 Fig — A. IL-6 does not have a differential effect on S phase distribution of bone marrow or splenic cells. Cells were isolated from 1 month old mice without the Eμ-myc transgene or with the transgene and the indicated IL6 genotypes [n = 6 for each genotype]. The percent of cells in S phase was measured by cell cycle analysis with propidium iodide staining and flow cytometry [FACSCalibur]. The error bars represent the standard deviation. B. Average tumor weights. Tumors formed in lymph nodes, thymi and spleens were isolated and weighed from moribund mice of the indicated genotypes [n>10 for each genotype]. The error bars represent the standard deviation. C. Representative moribund IL6+/+ and IL6-/-;Eμ-myc mice are shown. Dashed line circles reflect presence [in IL6+/+;Eμ-myc] or absence [in IL6-/-;Eμ-myc] of enlarged lymph nodes. Solid line circle demarks lymphoma in thymus of IL6-/-;Eμ-myc mouse. D. Histological examination [H&E staining] of lymphomas arising in Eμ-myc mice of the indicated IL-6 genotypes. Scale bars 100μM. (PDF) [file pone.0247394.s001.pdf]

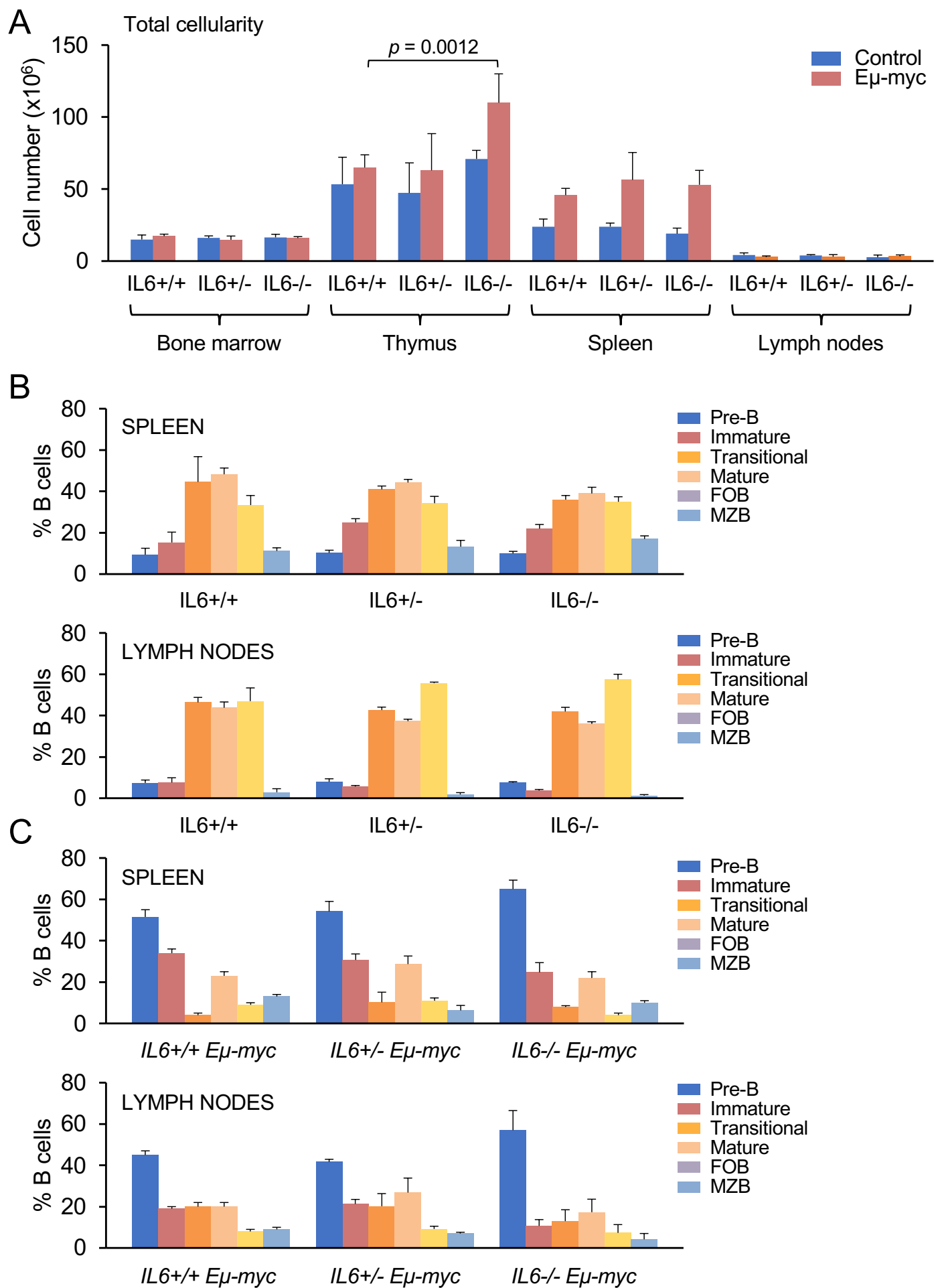

D

Pre-tumor Thymi (B cells)

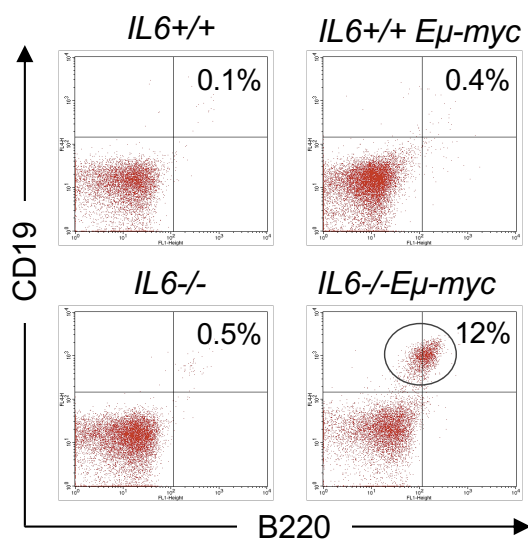

Thymic Tumors (B cells)

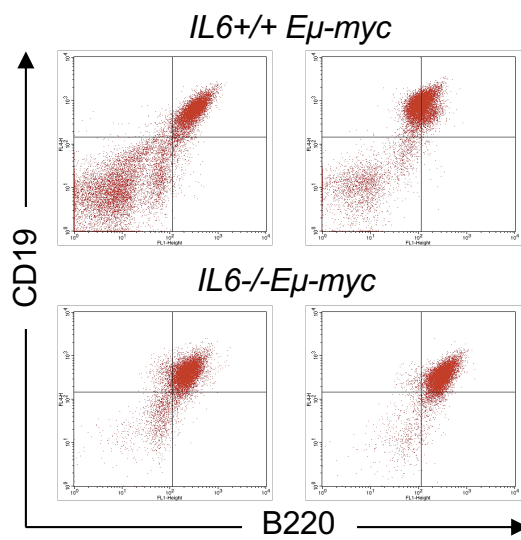

Pre-tumor Thymi (T cells)

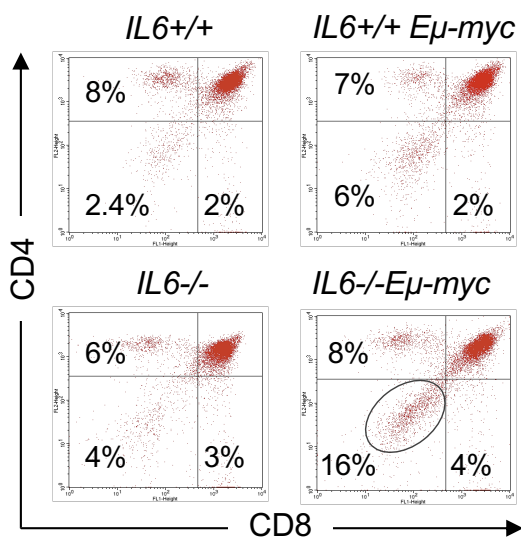

Pre-tumor Spleens (T cells)

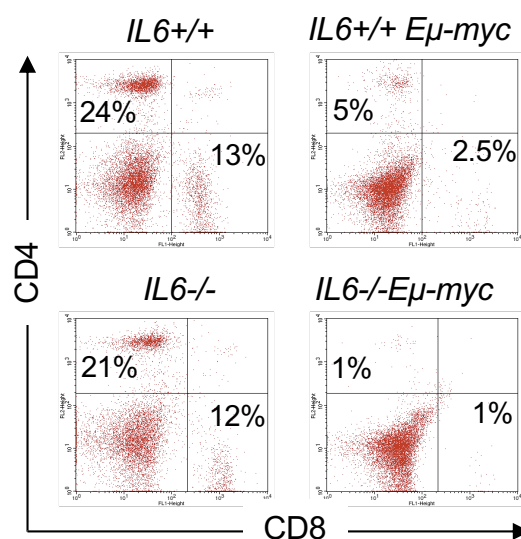

Supplement: S2 Fig — A. Total cellularity of primary and secondary lymphoid organs as noted of 1-month old control and Eμ-myc mice of the indicated IL-6 genotypes [n>10 for each genotype]. The error bars correspond to standard deviation. B. Flow cytometry analysis of B cells in the spleen and lymph nodes of 1-month old non-transgenic IL6+/+ [WT] and IL6-/- mice. Cell populations were defined as Pre-B [CD19+B220lowCD43-IgM-], immature B [CD19+B220lowIgM+], transitional B [CD19+B220highIgM+], mature B [CD19+IgM+IgD+]. follicular B [FOB] [B220+IgMlow/-IgD+] and marginal zone B [MZB] [B220+IgM+IgDlow/-] [78]. The results are representative of 6 to 8 mice of each genotype. The error bars correspond to standard deviation. C. Flow cytometry analysis of B cells in the spleen and lymph nodes of 1-month old IL6+/+; Eμ-myc and IL6-/-;Eμ-myc mice as in S2B. D. Representative flow cytometry scatter plots are shown of thymi or spleens from mice with specific genotypes. IL6-/- Eμ-myc mice showed elevated B cell numbers in pre-tumor thymi [top left]. IL6-/- and IL6-/- Eμ-myc mice showed increased immature double negative T cells in pre-tumor thymi [bottom left]. Eμ-myc mice had reduced CD4+ and CD8+ T cells in pre-tumor spleens, particularly evident in IL6-/- Eμ-myc mice. (PDF) [file pone.0247394.s002.pdf]

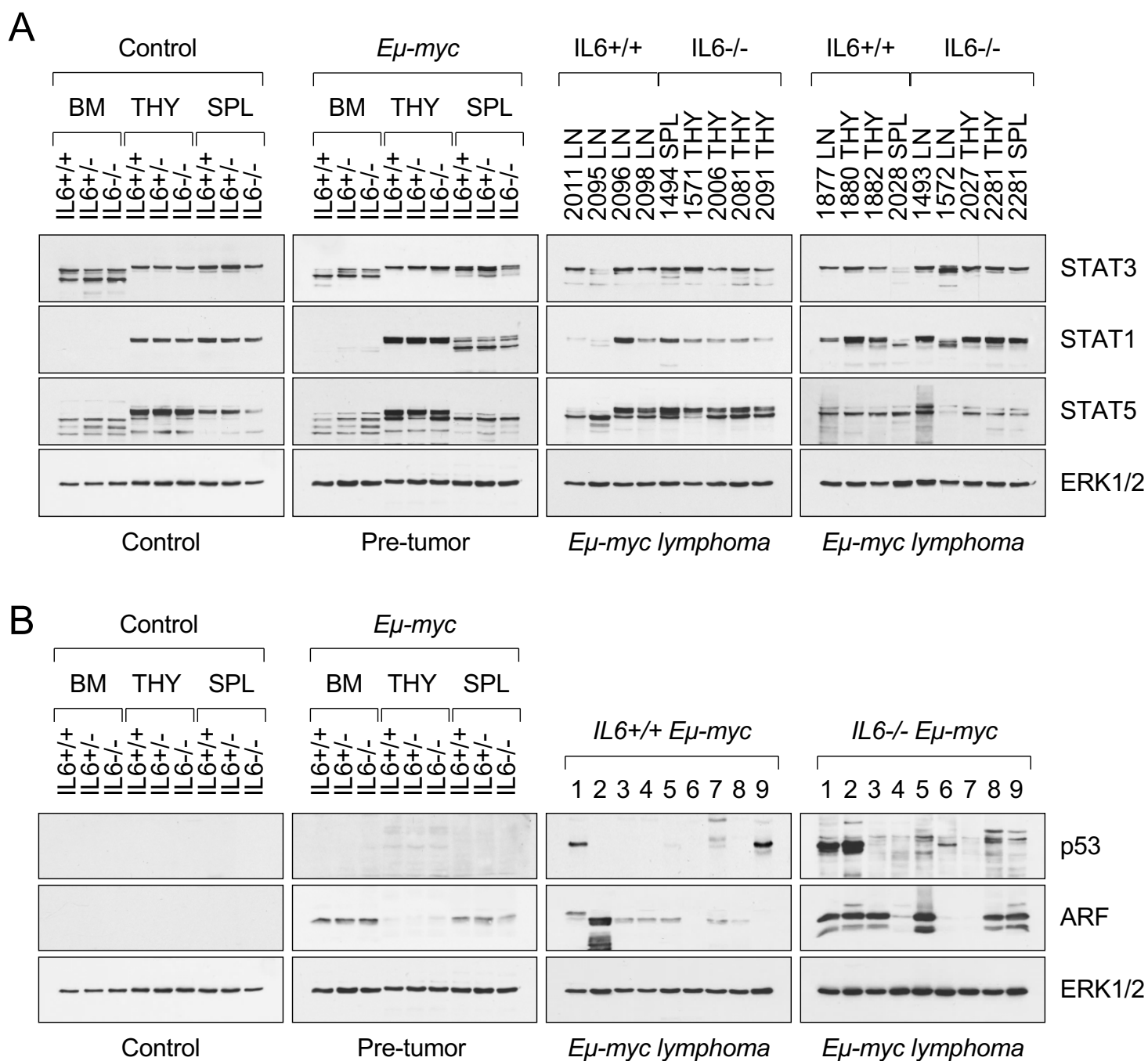

Supplement: S3 Fig — A. Western blot analysis of STAT3, STAT5, and STAT1 expression in bone marrow [BM], thymi [THY], and spleens [SPL] of individual control mice lacking the Eμ-myc transgene or pre-tumor [1 mo age], of Eμ-myc mice with the indicated IL-6 genotypes, and in B cell lymphomas derived from these mice. B. Western blot analysis of p53 and ARF expression in BM, THY and SPL of control mice lacking the Eμ-myc transgene, and of pre-tumor [1 mo age] Eμ-myc mice, and B cell lymphomas derived from these mice. (PDF) [file pone.0247394.s003.pdf]

A

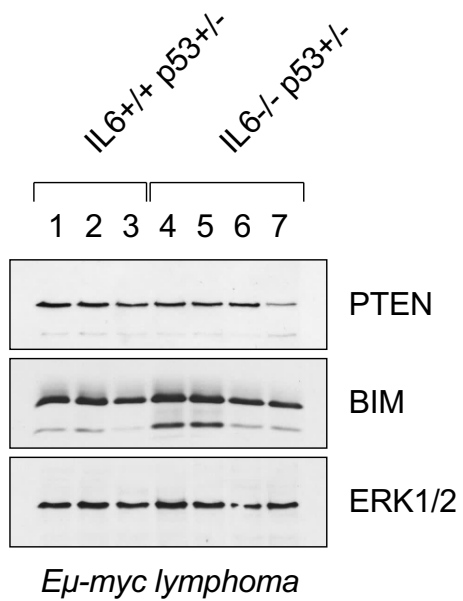

B

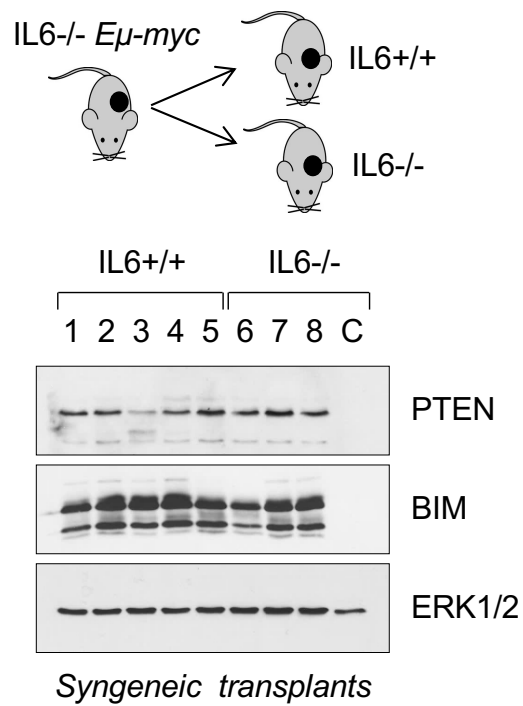

Supplement: S4 Fig — A. Western blot of PTEN and BIM expression in BM from individual mice IL6+/+;p53+/- Eμ-myc and IL6-/-;p53+/- Eμ-myc. B. Western blot analysis of PTEN and BIM expression in IL6-/-;Eμ-myc lymphomas developed from cell transplants into WT or IL6-/- syngeneic recipients. [C] corresponds to sample of bone marrow from a control mouse that received no transplant. (PDF) [file pone.0247394.s004.pdf]

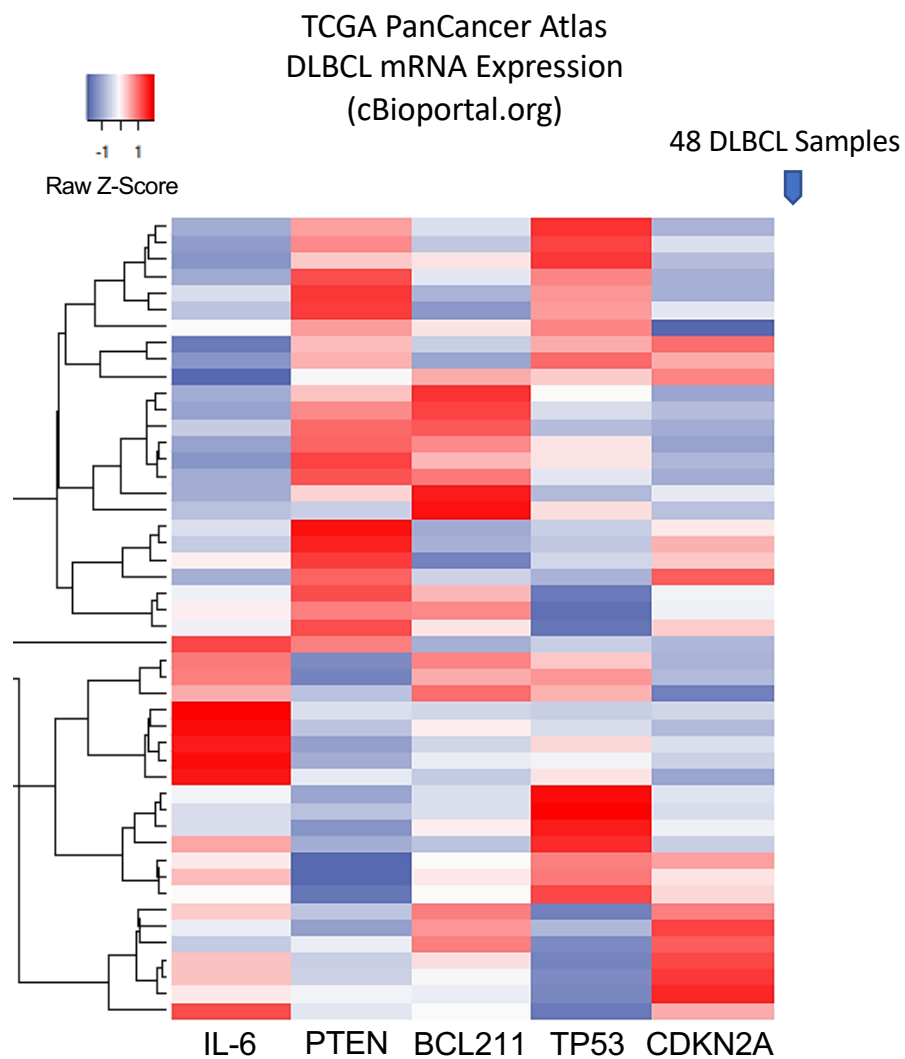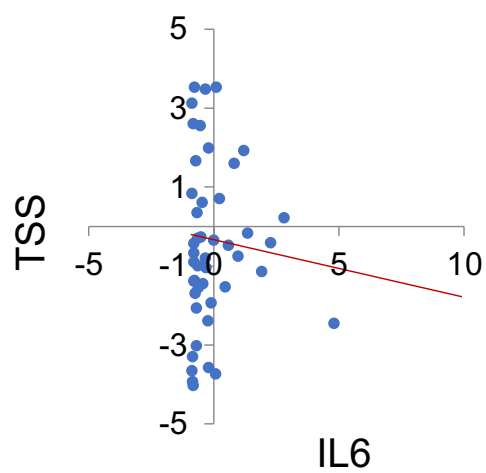

Supplement: S5 Fig — The heatmap displays mRNA expression of the IL-6 gene and the PTEN, BCL2L11, TP53, and CDKNA2 genes in 48 DLBCL samples. Z-scores relative to diploid samples [RNA Seq V2 RSEM] are presented [cBioportal.org] [heatmapper.ca]. A scatter plot comparing IL-6 expression with the sum of Z-scores of the four tumor suppressor genes [tumor suppressor signature, TSS] is shown for each sample. (PDF) [file pone.0247394.s005.pdf]

Fig 1C

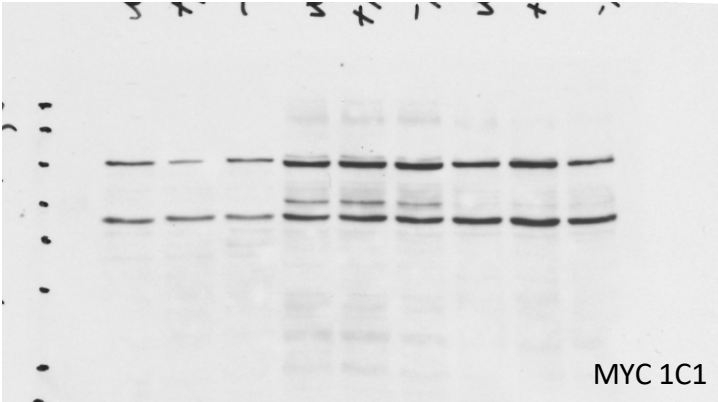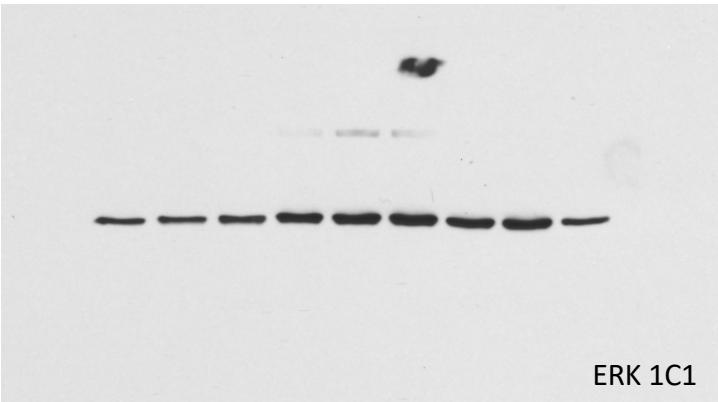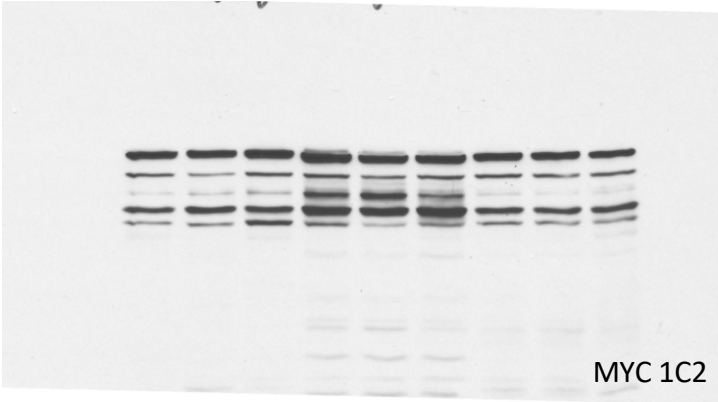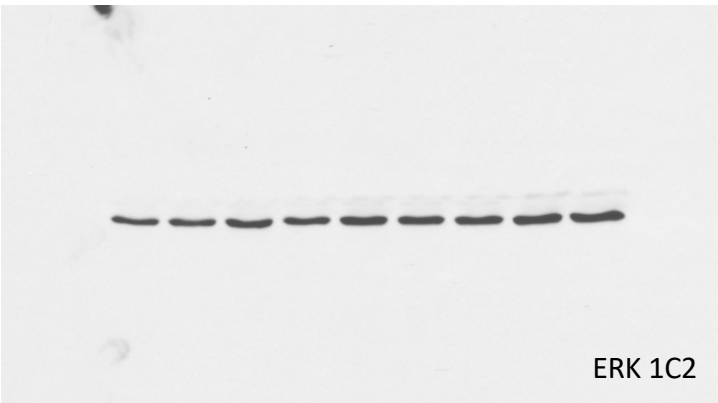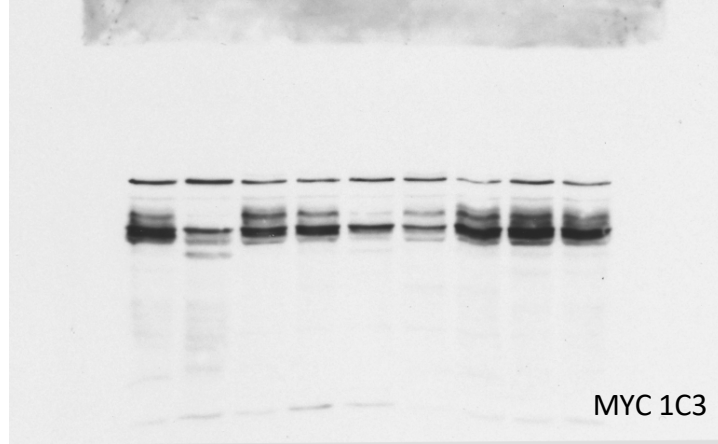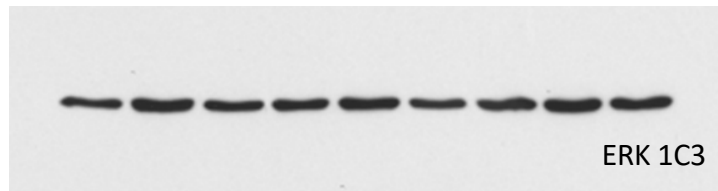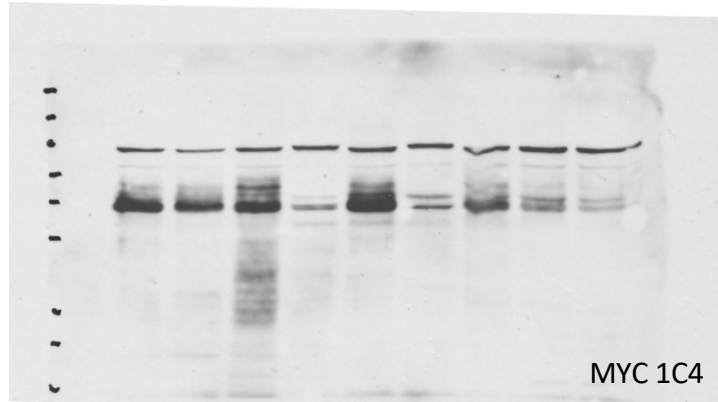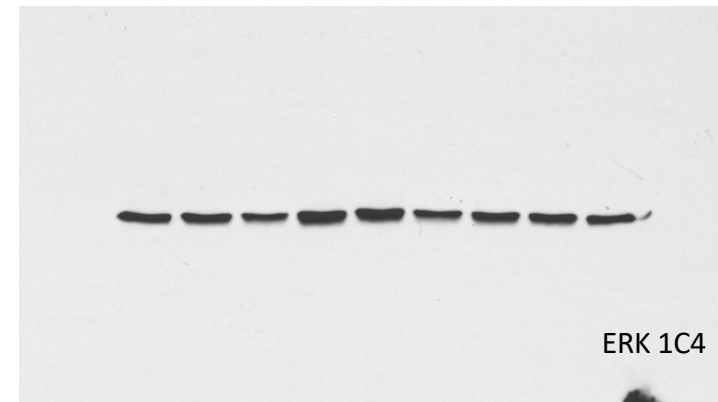

Fig 3 B

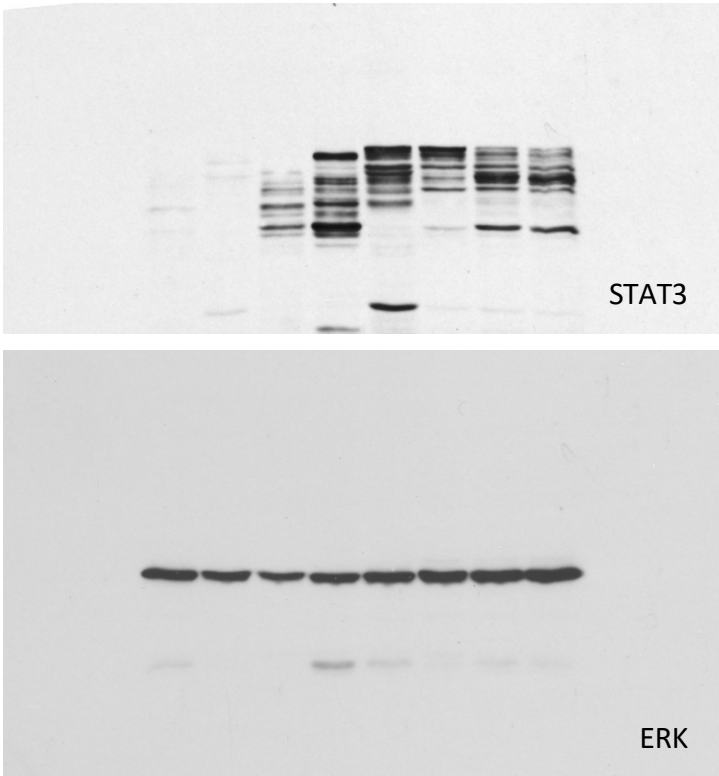

Fig 4A

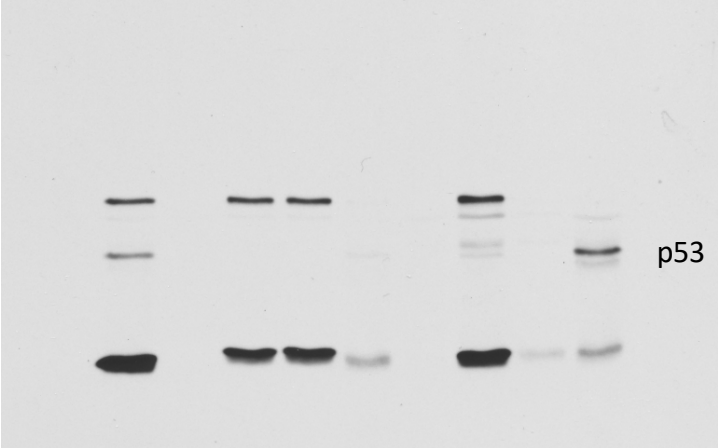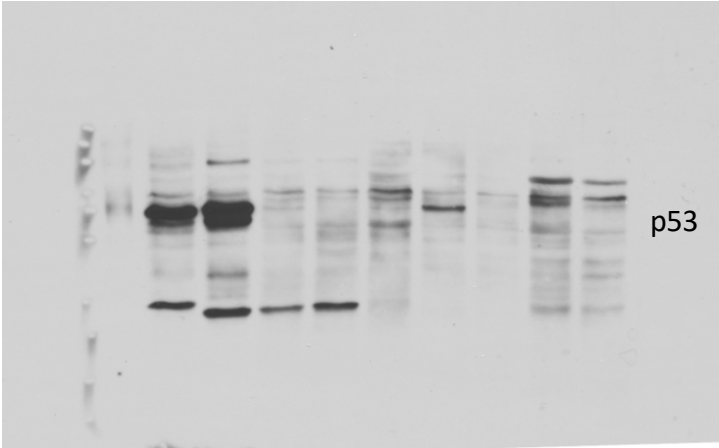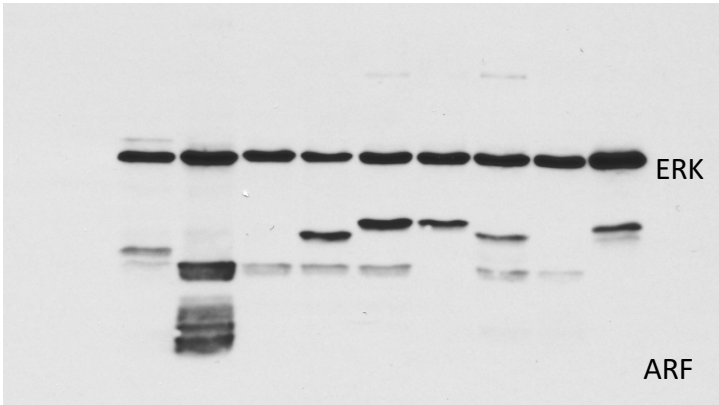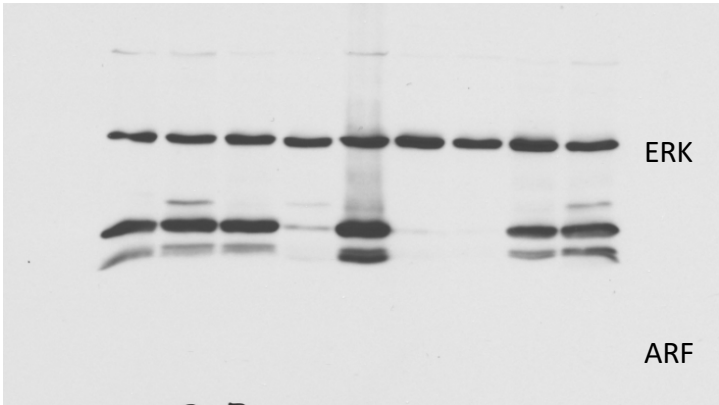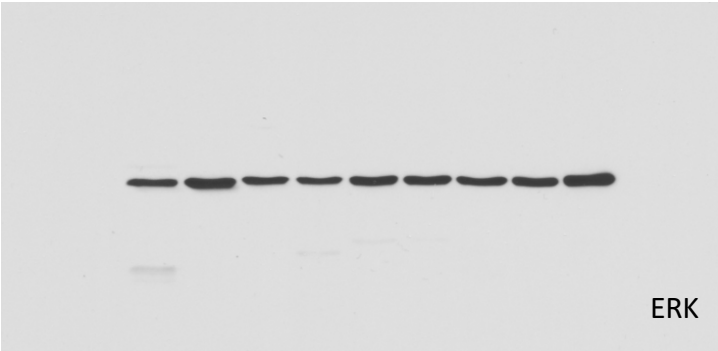

Fig 4C

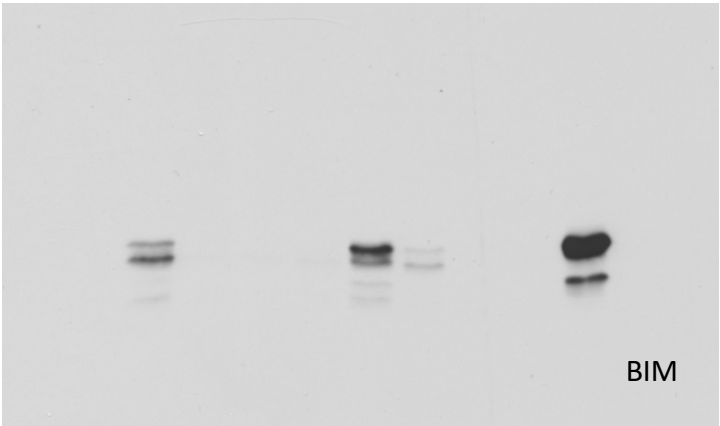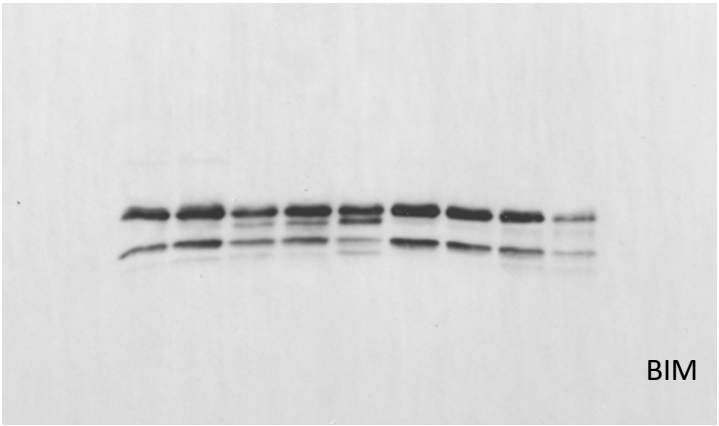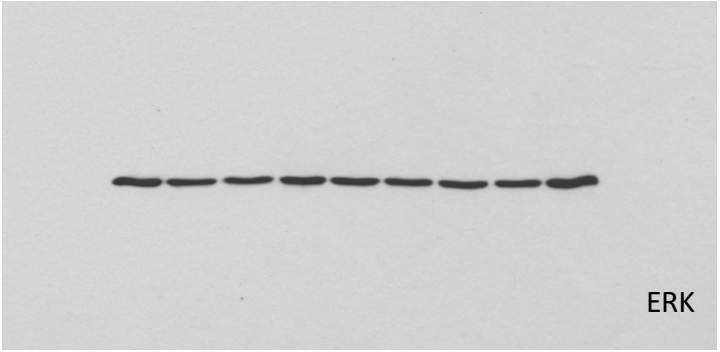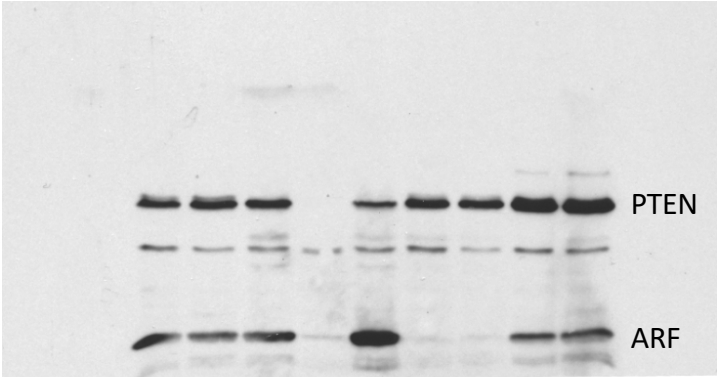

Fig S3A

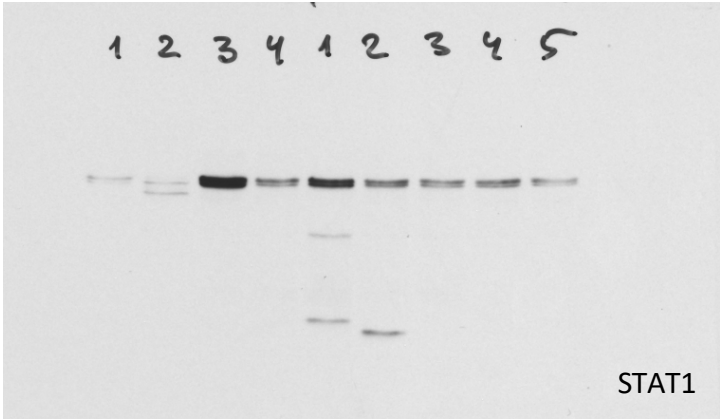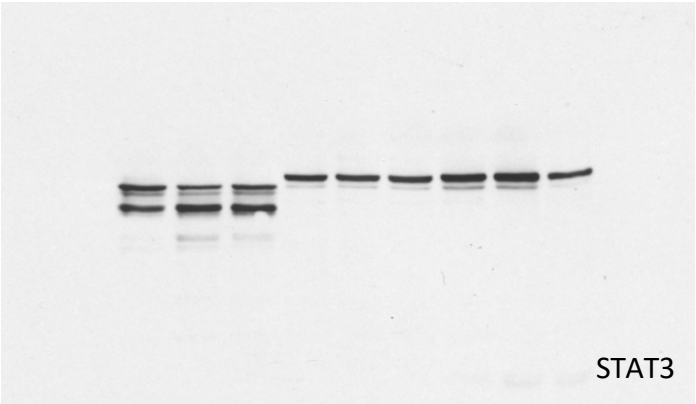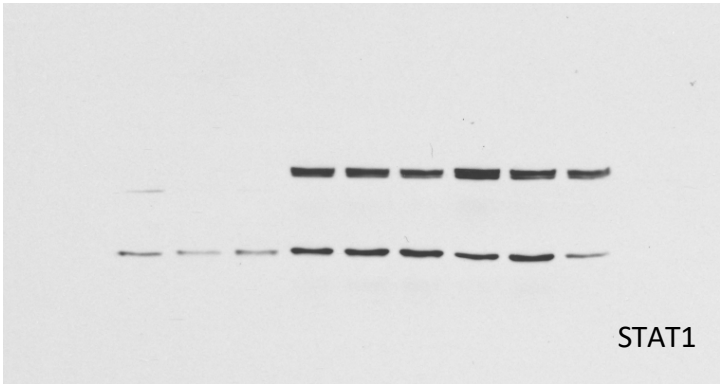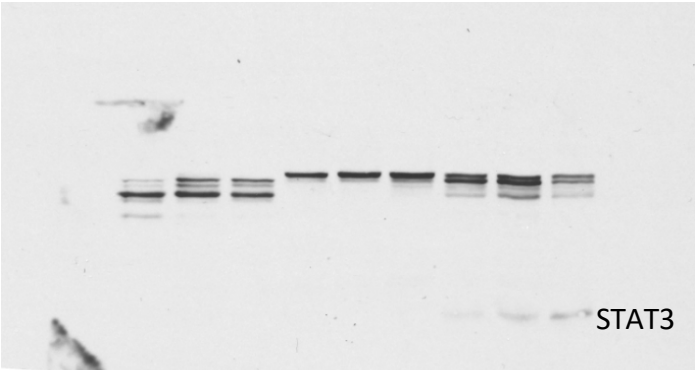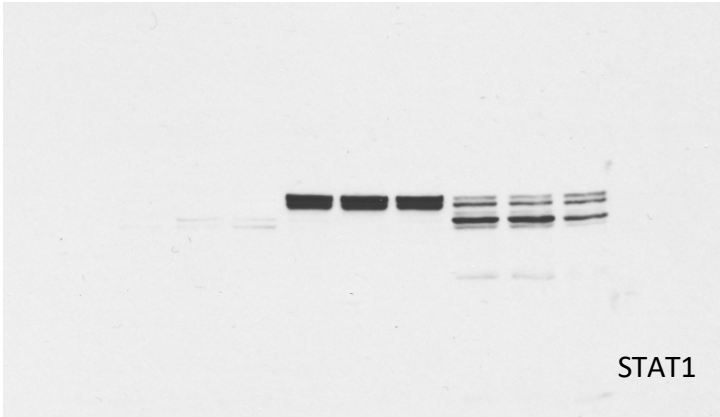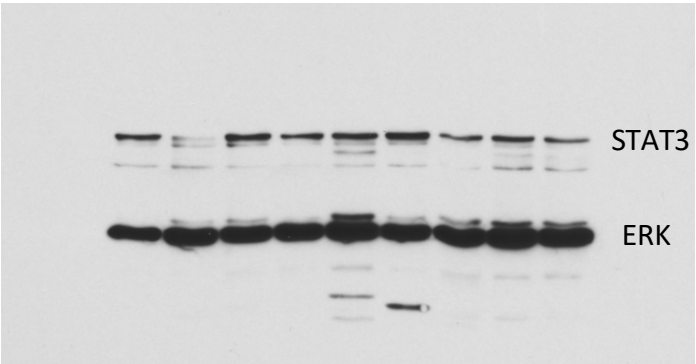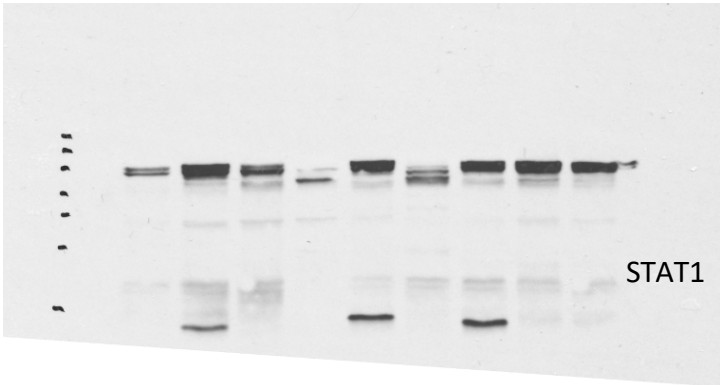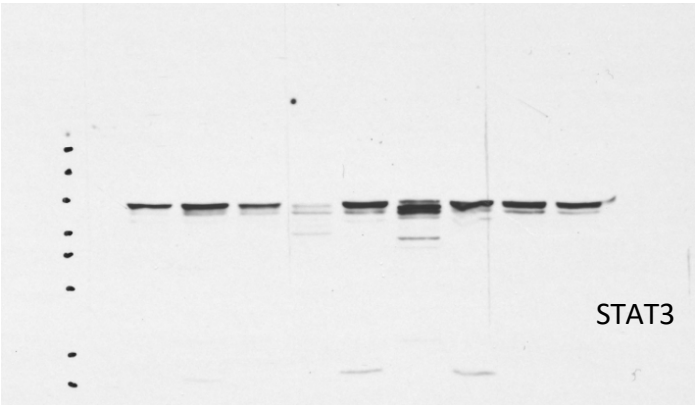

Fig S3A

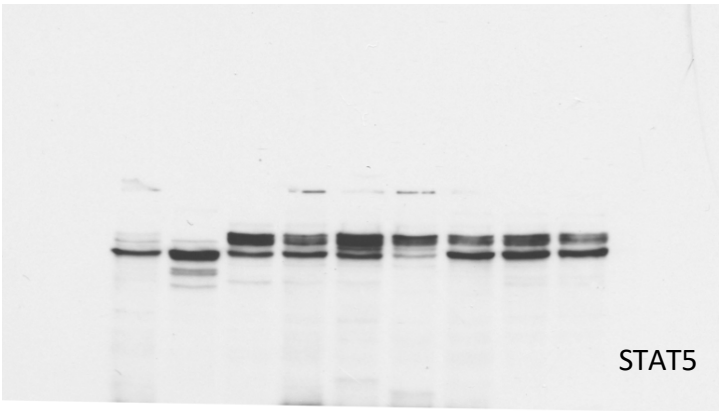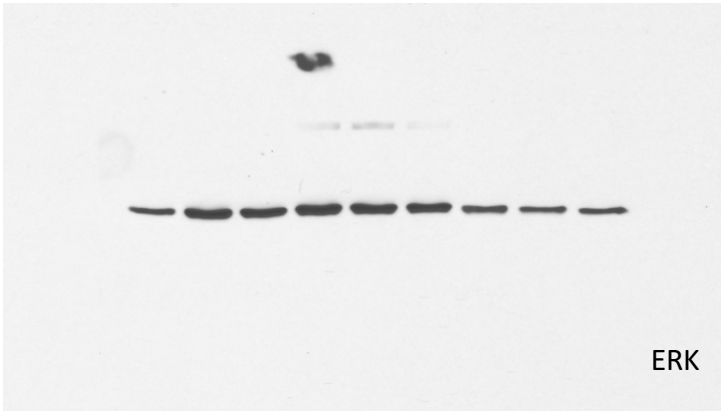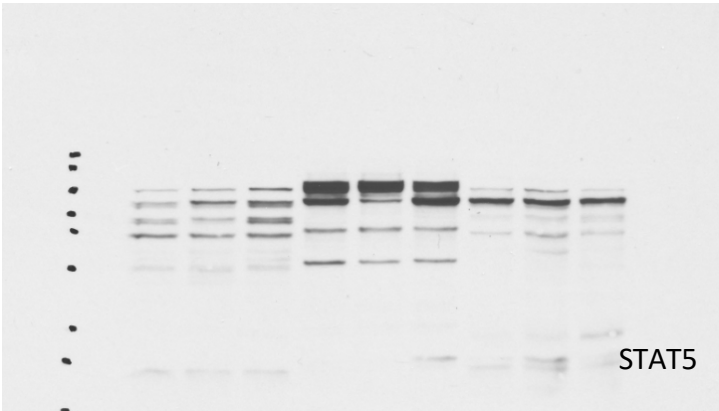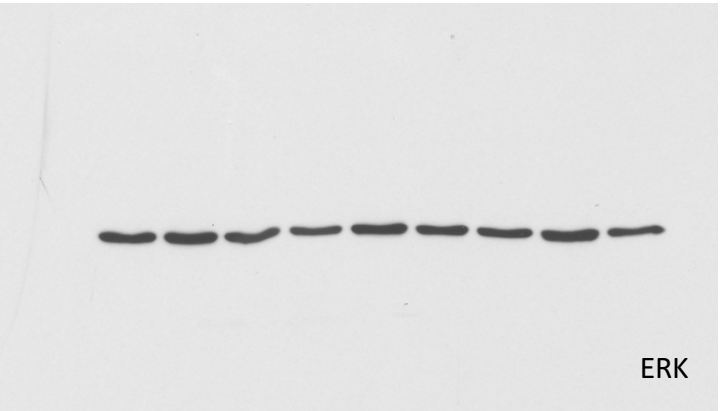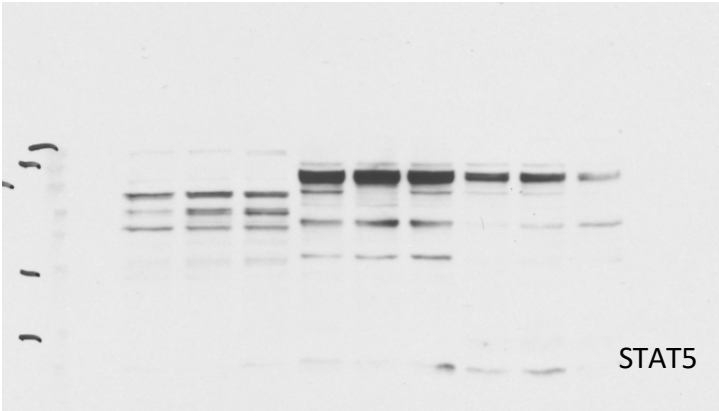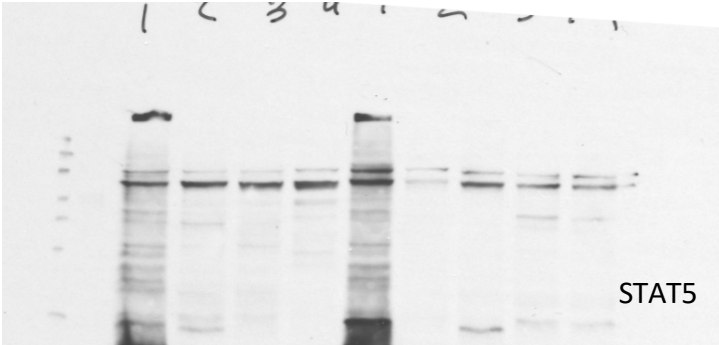

Fig S3B

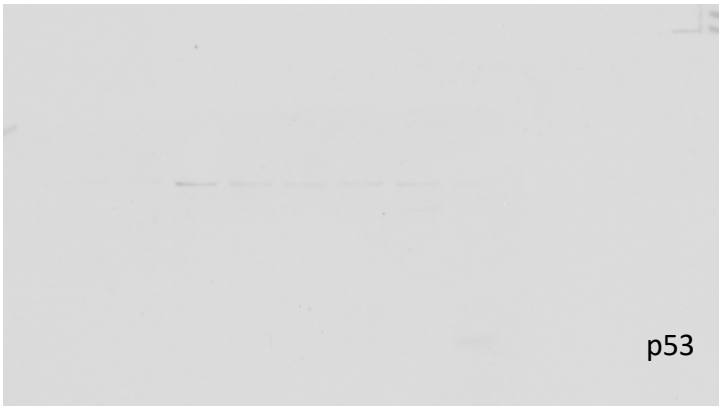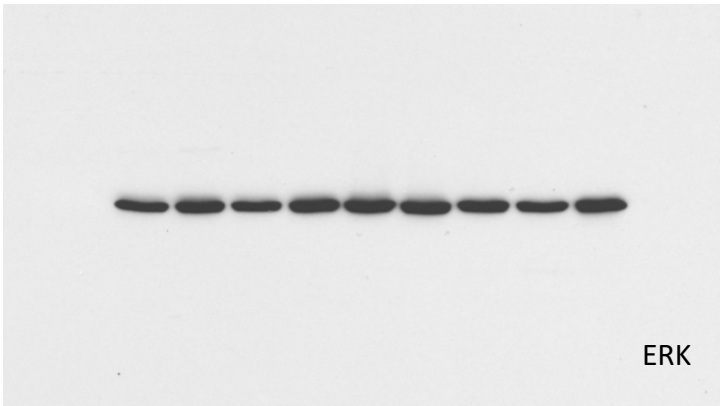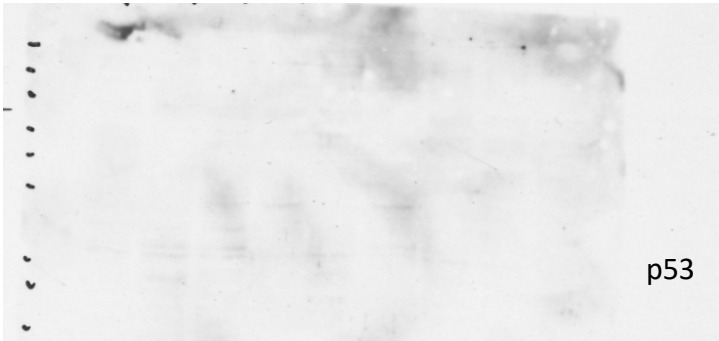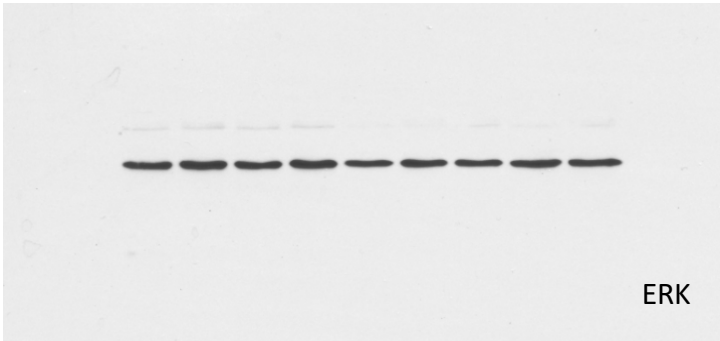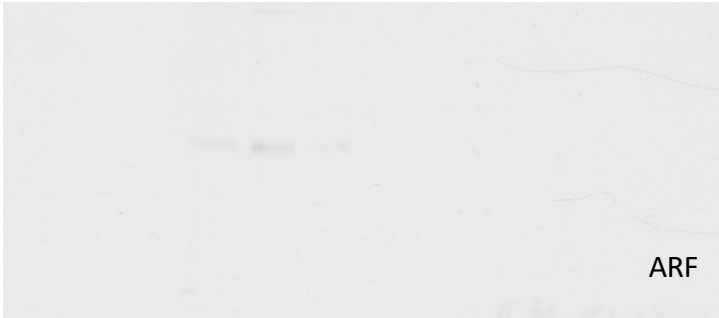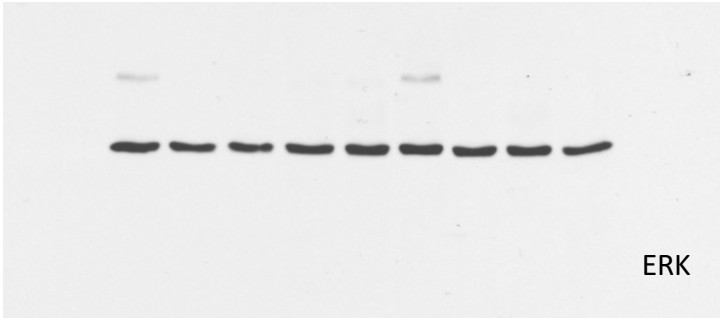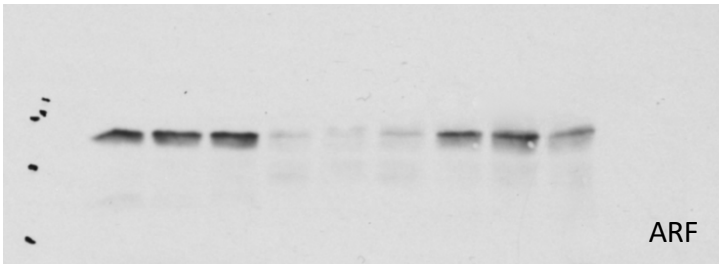

Fig S4A

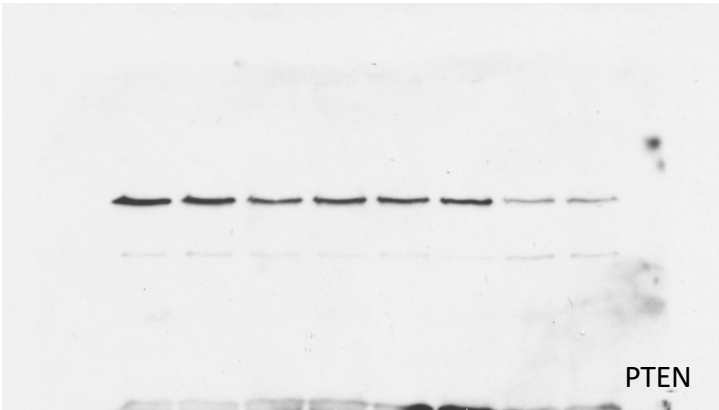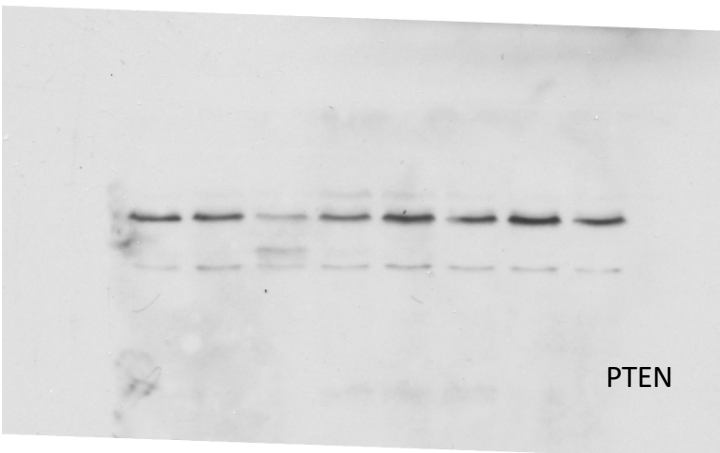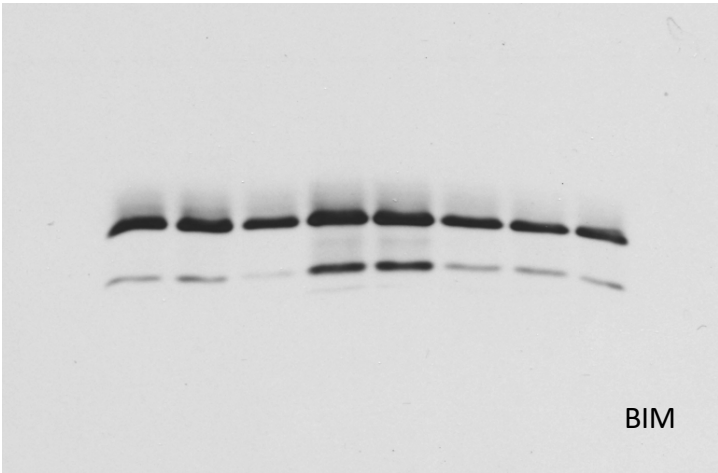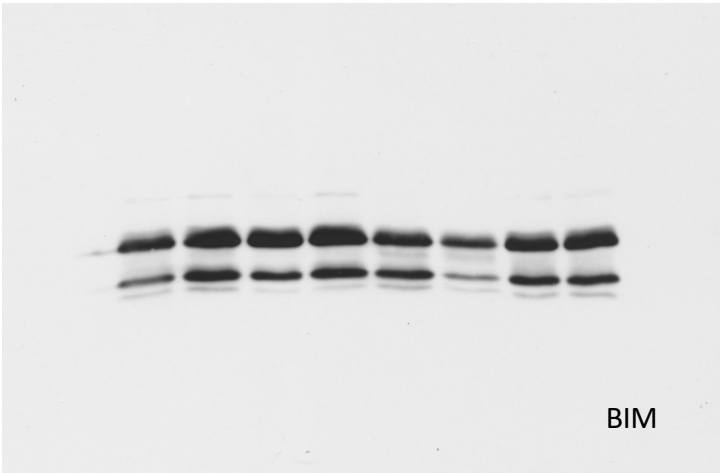

Fig S6B

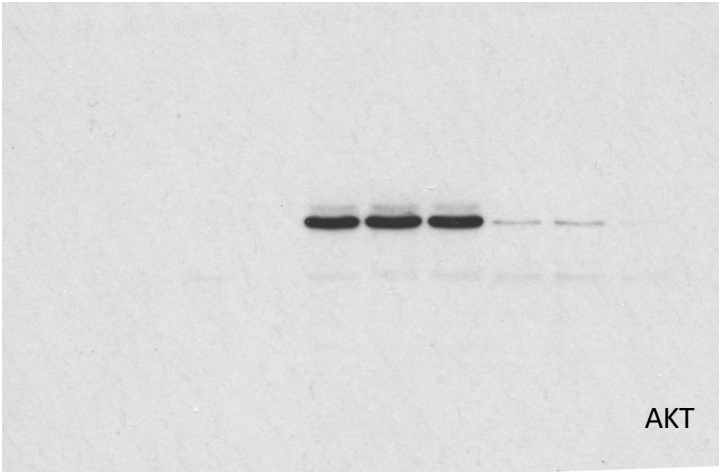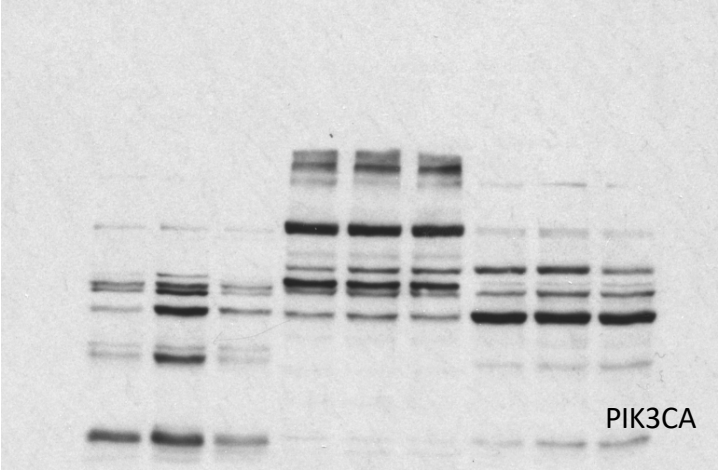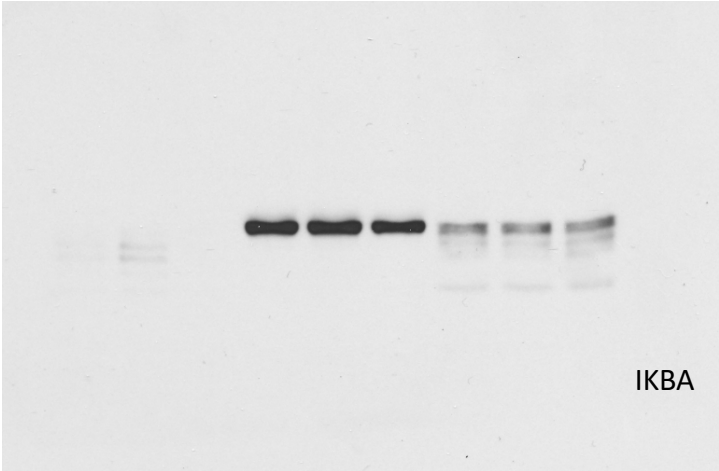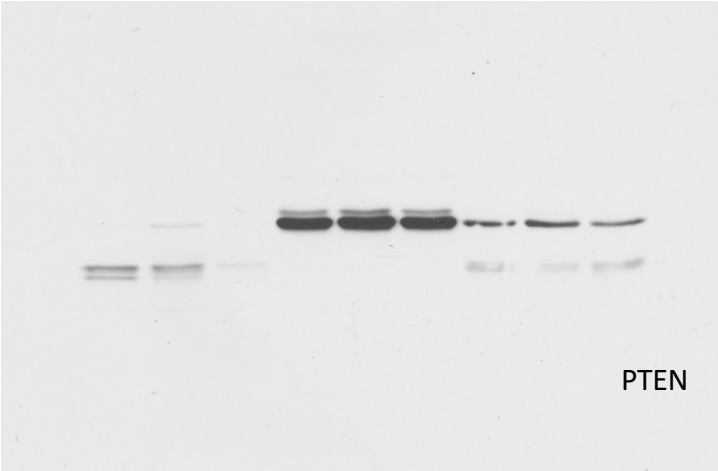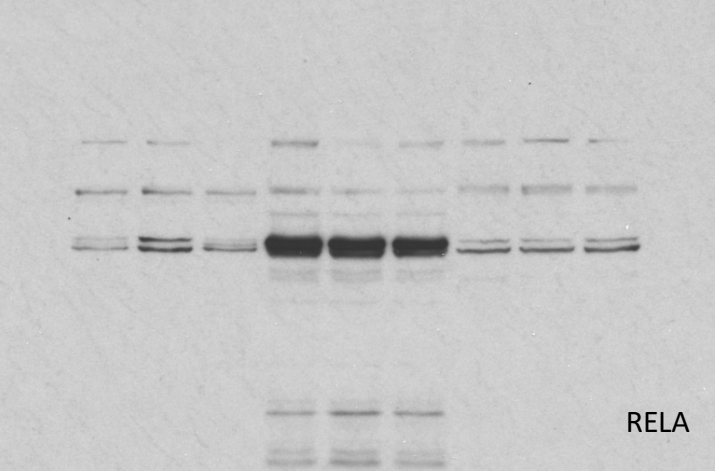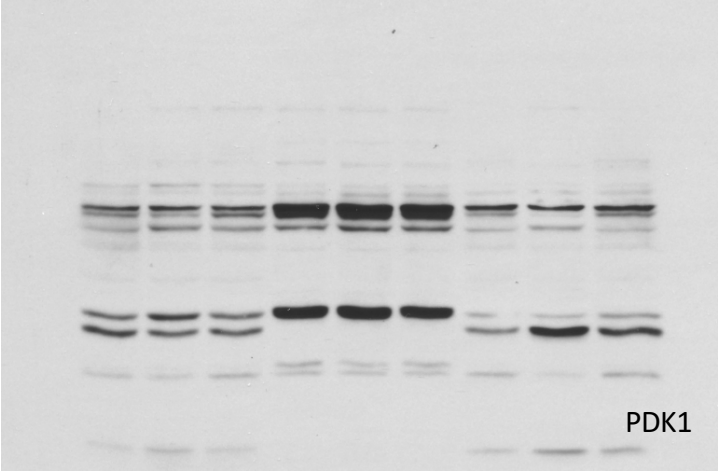

Fig S6B

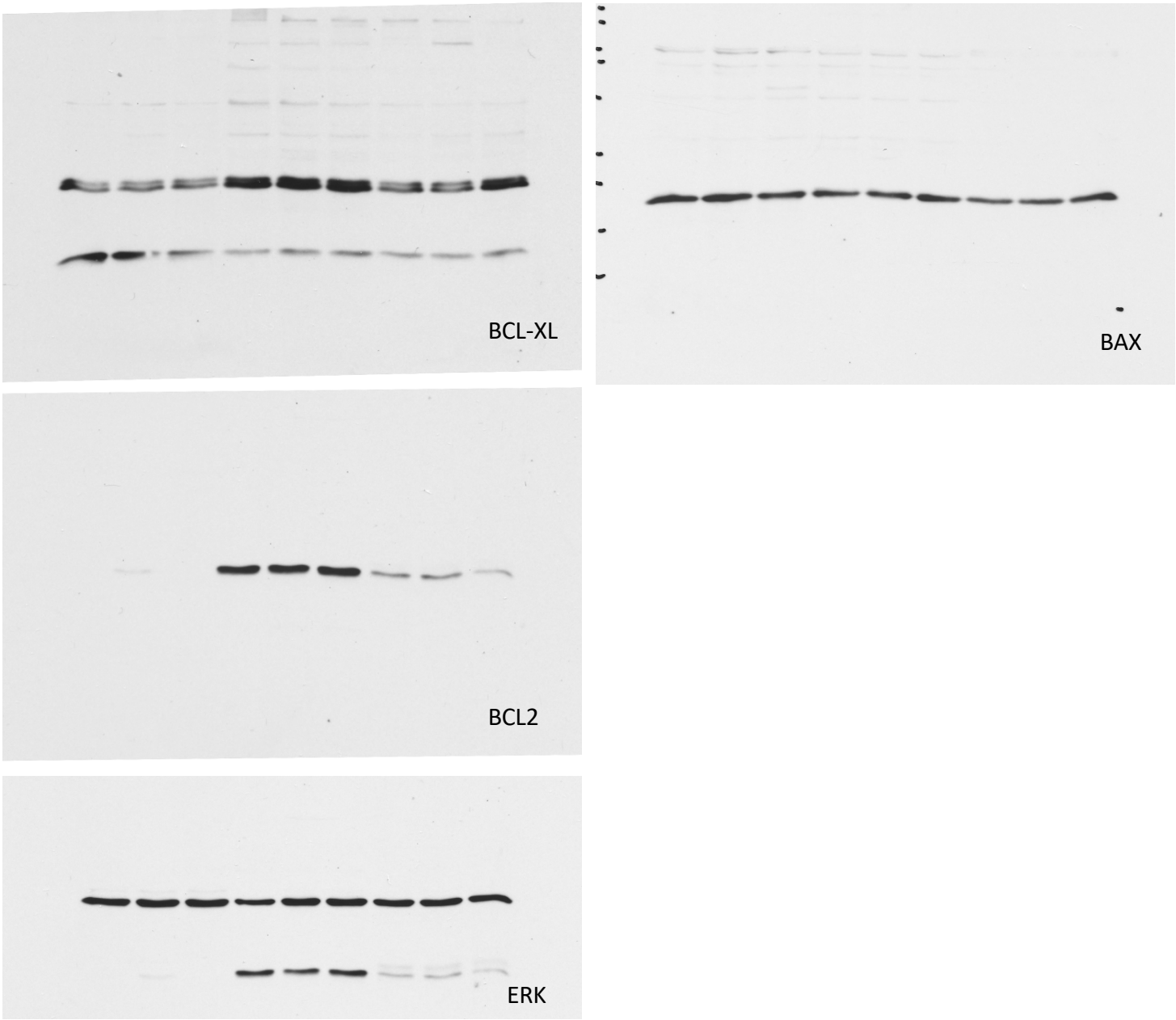

Fig S6D

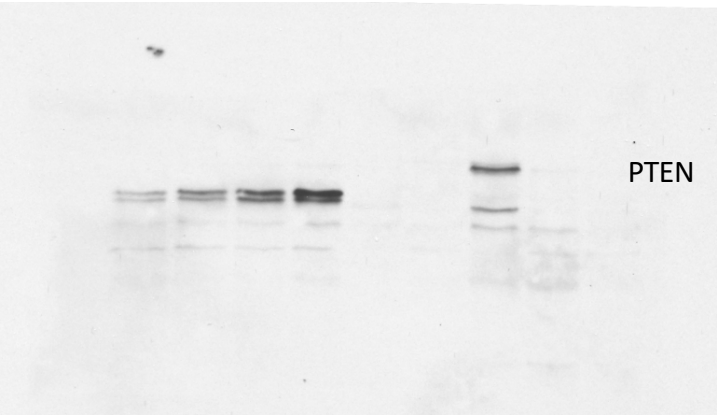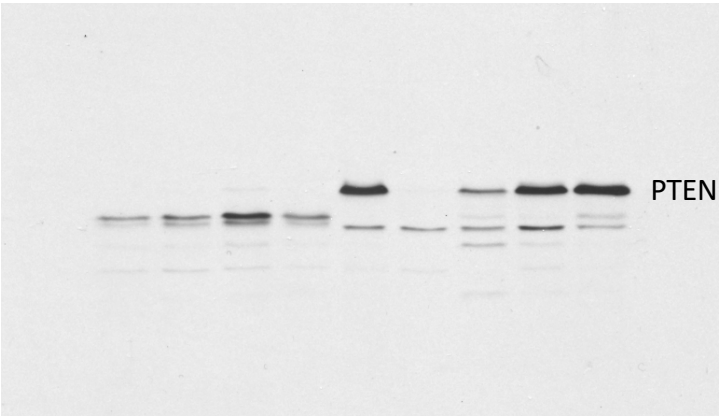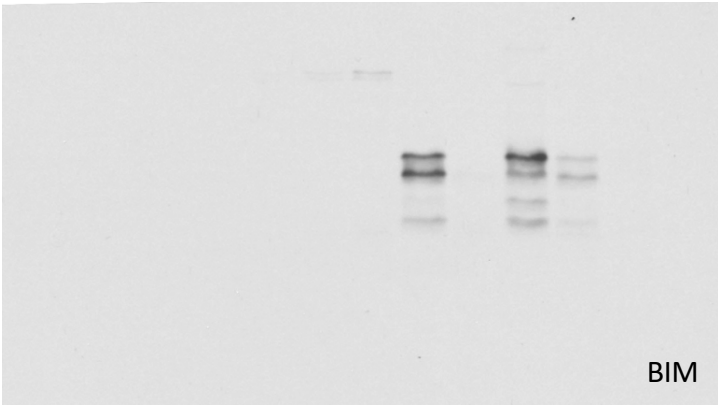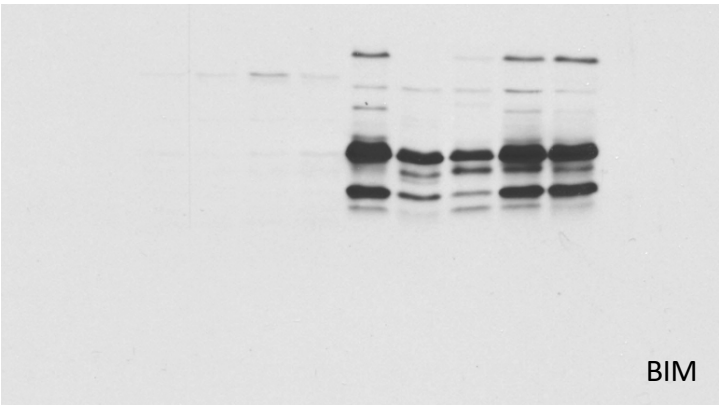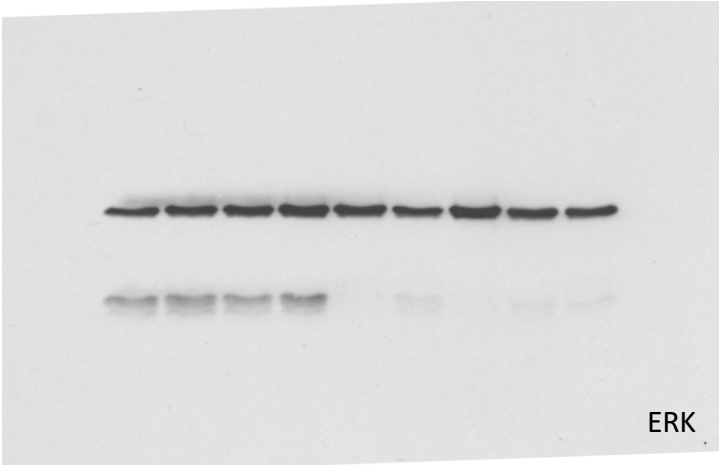

Supplement: S1 Raw images — (PDF) [file pone.0247394.s007.pdf]
