# Supplementary material for: IL-6 promotes MYC-induced B cell lymphomagenesis independent of STAT3
Source: PLoS One. 2021 Mar 2;16(3):e0247394. doi: 10.1371/journal.pone.0247394 (PMC7924759; doi:10.1371/journal.pone.0247394)
Supplement: S6 Fig — A. Western blot analyses of primary and secondary lymphoid organs of 1 month old Eμ-myc mice of the indicated IL-6 genotypes. B. Western blot of BM protein samples from a subset of the individual IL6+/+;Eμ-myc and IL6-/-;Eμ-myc mice that were pooled for the RPPA analyses. C. Western blot for PTEN and BIM in BM samples from pre-tumor mice and tumor-bearing IL6+/+;Eμ-myc and IL6-/-;Eμ-myc mice. (PDF) [file pone.0247394.s006.pdf]

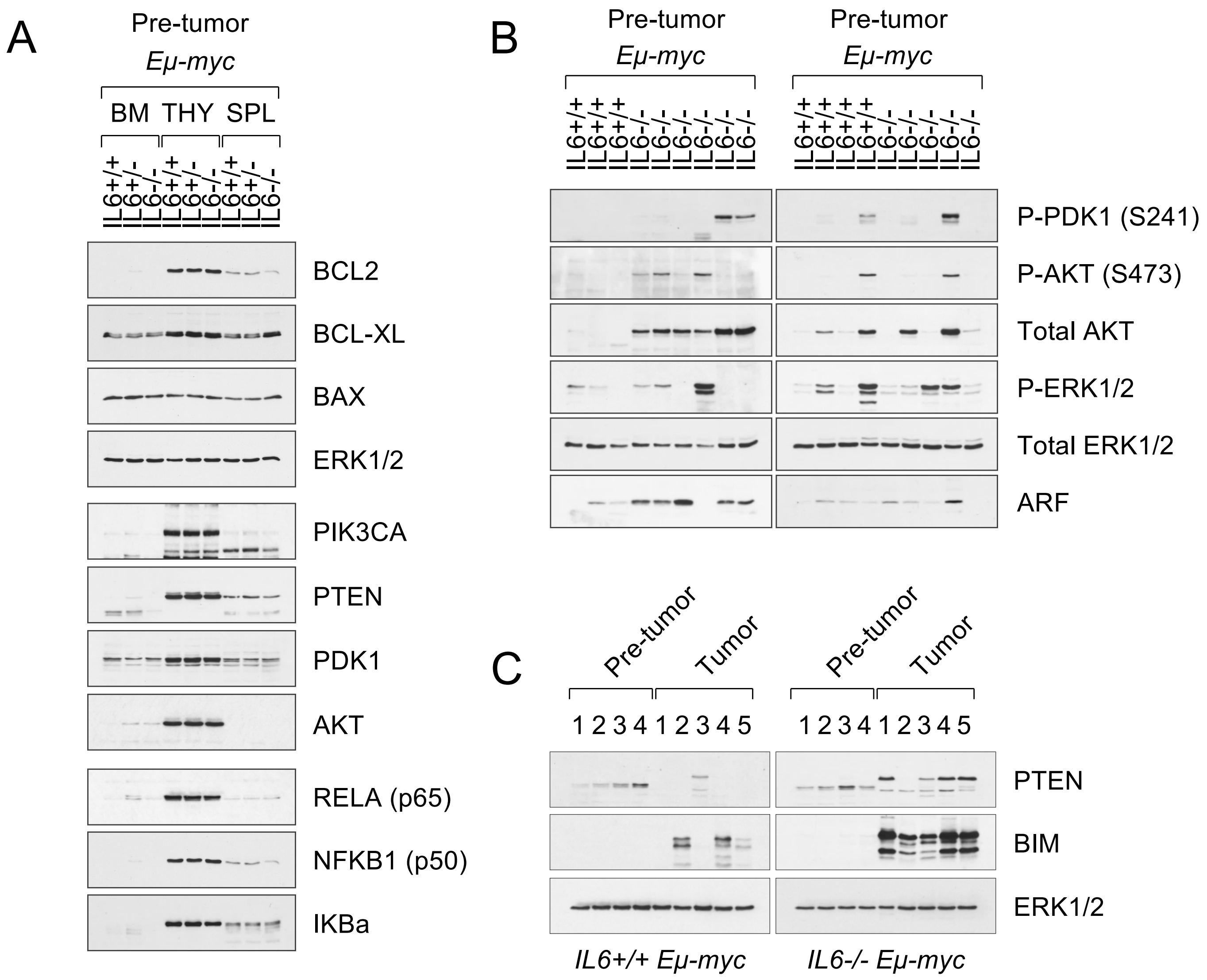
A
Pre-tumor
Eμ-myc
BM THY SPL
IL6+/+
IL6+/-
IL6-/-
BCL2
BCL-XL
BAX
ERK1/2
PIK3CA
PTEN
PDK1
AKT
RELA (p65)
NFKB1 (p50)
IKBa
B
Pre-tumor
Eμ-myc
P-PDK1 (S241)
P-AKT (S473)
Total AKT
P-ERK1/2
Total ERK1/2
ARF
C
Pre-tumor
Tumor
1 2 3 4 1 2 3 4 5
PTEN
BIM
ERK1/2
IL6+/+ Eμ-myc
IL6-/- Eμ-myc
